# CH_4_ Decomposition on Nickel Phyllosilicate: Switching from Tip to Base Growth of Carbon Nanotubes

**DOI:** 10.1002/smll.202500994

**Published:** 2025-06-04

**Authors:** Esteban Gioria, Vivianne K. Ocampo‐Restrepo, Anton Simon Bjørnlund, Verdande Kim Pedersen, Stig Helveg, Ib Chorkendorff, Christian Danvad Damsgaard

**Affiliations:** ^1^ Department of Physics Technical University of Denmark Lyngby 2800 Kgs Denmark; ^2^ Center for Visualizing Catalytic Processes (VISION) Department of Physics Technical University of Denmark Lyngby 2800 Kgs Denmark; ^3^ National Centre for Nano Fabrication and Characterization (Nanolab) Technical University of Denmark Lyngby 2800 Kgs Denmark

**Keywords:** base growth, carbon nanotubes, methane decomposition, nickel, phyllosilicate

## Abstract

The emerging trends in carbon nanotube applications make them exceptional functional materials of highly added value. Thermocatalytic CH_4_ decomposition is an effective pathway toward their production, forming H_2_ as the only byproduct. However, catalyst deactivation due to sintering and blockage of the active sites, together with their detachment from the support remains a challenge. In this work, nickel phyllosilicate is employed as a catalyst precursor for the formation of active and stable metal sites. Surprisingly, the particles remain attached to the support, switching from the typical tip‐growth reported for state‐of‐the‐art catalysts to a base growth mechanism. The nickel nanoparticles remain stable against sintering even under harsh conditions up to 750 °C. A combination of DFT calculations, in situ TEM, and in situ XRD studies reveals that the reduction of Ni─O bonds, particularly those involving silicon‐bonded oxygen (Si─O─Ni; apical oxygen), requires high temperatures. Post‐activation, the small, dispersed nickel nanoparticles catalyze CH_4_ decomposition into carbon nanotubes and H_2_. Unlike prior reports, in situ XRD confirms no nickel carbide formation in the bulk. Additionally, in contrast to any known nickel‐based catalyst, it is demonstrated that particles below 10 nm can effectively activate CH_4_ cracking, avoid encapsulation, and enable the base‐growth of micrometer‐long, narrow carbon nanotubes.

## Graphical abstract

Nickel phyllosilicate is an excellent catalyst precursor for CH_4_ decomposition, forming small and strongly anchored nickel nanoparticles that promote base‐growth of carbon nanotubes and hydrogen production. The catalyst resists sintering at even 750 °C. In situ XRD and TEM characterization, together with DFT calculations, indicate that Ni─O─Si bond reduction is responsible for the high stability. Unlike conventional impregnated catalysts, sub‐10 nm particles remain active without encapsulation, and nickel carbide phase formation is discarded.

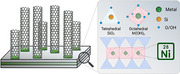

## 1 Introduction

Carbon nanotubes (CNTs) are a significant class of functional material with remarkable properties at the nanoscale. Their high strength, lightweight structure, electrical conductivity, and high thermal stability make them suitable for a wide range of applications in electronics, energy storage, structural materials, and even healthcare.^[^ ^1^, ^2^, ^3^ ^]^ For example, their high conductivity compared to traditional materials, makes them vital in the development of faster and smaller electronic devices.^[^ ^4^ ^]^ Similarly, CNTs enhance the strength and durability of composite materials while keeping them lightweight. This has made them highly useful in the aerospace, automotive, and sporting goods sectors.^[^ ^5^ ^]^ Similarly, and in line with the energy transition, the application of CNTs in energy storage and conversion technologies like batteries, capacitors, and fuel cells is a growing field.^[^ ^6^, ^7^, ^8^ ^]^ As a highly added‐value product, it is expected that the global market of CNTs will experience significant growth. In 2021, the CNTs market was valued at USD 5 billion, with a projected growth rate of 14–18% within the next decade.^[^ ^1^ ^]^ This is in accordance with the growing market of end‐use applications.

CNTs are generally produced by chemical vapor deposition (CVD), due to simplicity, scalability, and cost. In CVD, carbon sources like ethylene (C_2_H_4_) or acetylene (C_2_H_2_) are thermally decomposed at high temperatures in the presence of catalysts, usually based on Fe, Co, or Ni. Despite the effectiveness of the method, it has a significant CO_2_ positive footprint of 600 tons of CO_2_ per ton of carbon product.^[^ ^9^ ^]^ Furthermore, CNTs are usually containing a certain fraction of the metallic phase, typically at the end tip of the nanostructure.^[^ ^10^, ^11^ ^]^ This is known as the tip‐growth mechanism and is very common for supported catalysts based on metal nanoparticles. After CNTs production, expensive and toxic purification treatments are applied for metal removal. For example, certain acids like HCl or HNO_3_ are employed for dissolving the metal nanoparticles and recovering the purified CNTs.^[^ ^12^ ^]^

In this context, catalytic CH_4_ decomposition (CMD) has gained attention since high purity H_2_ and carbon are produced as only products. Moreover, the process is industrially appealing due to its reliance on a low‐cost carbon source and the availability of well‐established processing infrastructure and distribution technologies.^[^ ^13^, ^14^ ^]^ CH_4_ decomposition is an endothermic reaction carried out at atmospheric pressure and temperatures between 500–900 °C. Despite metal supported catalysts based on abundant Fe, Co, and Ni shows high activity, CMD presents numerous challenges impeding the process development at the commercial scale.^[^ ^13^ ^]^ CH_4_ requires a higher energy per mole to decompose than traditional C_2_H_2_ or C_2_H_4_, due to the strong stability of single C─H bonds. High‐temperature conditions and C─C bond formation promote catalysts deactivation due to sintering and/or blockage of the active sites due to carbon encapsulation. A promising alternative was recently reported by Chen et al., who employed a NiMo‐Bi liquid alloy in its molten state.^[^ ^15^ ^]^ While the system demonstrated excellent stability and high activity for H₂ production, the morphology of the resulting carbon products was not characterized.

In general terms, C‐H activation takes place on metallic sites. Once the active site becomes saturated with carbon, carbon precipitates on the surface, leading to the formation of CNTs. Depending on the growth mechanism, CNTs can promote growth by the detachment of the metal sites (known as tip‐growth) or following tangential growth (known as base‐growth) and remain attached to the support.^[^ ^10^, ^16^ ^]^ From a commercial perspective, the formulation of catalysts that promote the base‐growth of CNTs is imperative, allowing for regenerable catalysts and suppressing the typical detachment of metal particles. Moreover, it is critical to use inexpensive transition metals and synthesis protocols that are reproducible and easy to scale‐up.

The primary factor governing the tip‐ versus base‐growth mechanism of CNTs is the metal‐support interaction. It should not be too weak, which leads to detachment of the active site, nor too strong, which inhibits the activation of the transition metal into active nanoparticles. In this context, metal phyllosilicates are a very interesting type of layered structure formed by a layer of transition metal in octahedral coordination between two SiO_2_ layers in tetrahedral coordination. These materials, particularly nickel phyllosilicates (Ni‐PS), have been proven to be excellent precursors due to the enhanced stability of the active sites formed after reduction (Ni‐PS‐Red).^[^ ^17^, ^18^ ^]^

Ni‐PS‐Red was mainly studied for CO_2_ methanation, CH_4_ partial oxidation, ethanol reforming, and biomass valorization.^[^ ^19^, ^20^, ^21^, ^22^, ^23^, ^24^ ^]^ Surprisingly, there are very few reports that employed Ni‐PS‐Red as a catalyst for methane decomposition.^[^ ^25^, ^26^, ^27^, ^28^ ^]^ The first studies were reported by J. Bitter et al., showing tip‐growth CNTs without providing information on the dynamics of the active site under reaction conditions.^[^ ^25^, ^26^ ^]^ Recently, Kawi et al. demonstrated that Ni‐PS based catalysts can be employed for CH_4_ decomposition at a remarkably low temperature of 500 °C with high yield up to 5.3 mol H_2_ g_cat_^−1^h^−1^. However, like previous findings, only tip‐growth CNTs with detached nickel nanoparticles and a broad diameter distribution were observed.^[^ ^27^ ^]^ Recently, Lee et al. suggested the formation of base‐growth CNTs on Ni‐PS‐Red.^[^ ^29^ ^]^ However, the transmission electron micrographs reveal CNT structures with open ends. Additionally, their study primarily relies on post‐mortem characterizations, with limited insights into the nature and evolution of the active site under dynamic reaction conditions.

Nickel‐based catalysts have been extensively studied for CH_4_ decomposition. Nevertheless, the exact nature of the active site under reaction conditions remains a subject of debate.^[^ ^14^ ^]^ Some authors claim the formation of a NiC_X_ carbide phase as a stable intermediate.^[^ ^29^, ^30^, ^31^ ^]^ Other studies have given direct evidence that metallic Ni is the active phase, and graphene growth is driven by surface or subsurface C diffusion. In this mechanism, monoatomic Ni step edges at the C–Ni interface restructures by incorporating diffusing carbon atoms, while Ni atoms are expelled and migrate to the nanoparticle front, ultimately resulting in tip‐growth of CNTs.^[^ ^32^, ^33^, ^34^ ^]^ Nevertheless, other studies are based on characterization post‐mortem and/or the use of other carbon sources like CO or C_2_H_4_.^[^ ^35^, ^36^, ^37^ ^]^

In the present work, we show that nickel phyllosilicates are excellent precursors for the formation of small and dispersed nickel nanoparticles that serve as active sites for the base growth of carbon nanotubes. In situ X‐ray diffraction (XRD) and in situ transmission electron microscopy (TEM) evidenced the nature and formation of the metal nanoparticles. The chemical structure of 2:1 nickel phyllosilicate is studied by density functional theory (DFT) calculations. The thermodynamic analysis of the Ni─O bond reduction suggests that the reduction of oxygen (O) atoms bonded to silicon (Si), known as apical oxygen, could limit the growth of nickel nanoparticles. Moreover, in situ XRD studies unravel the formation of CNTs and the absence of NiC_X_ species in the bulk under dynamic reaction conditions. The comparison of Ni‐PS catalyst with state‐of‐the‐art impregnated Ni/SiO_2_ proves that the strong metal‐support interaction between the nickel sites and the silica structure gives rise to small and strongly attached nickel particles that completely switch the mechanism from a typical tip‐growth (for the impregnated catalyst) to a base growth for the nickel phyllosilicate.

These findings are of great relevance for the understanding of CH_4_ decomposition onto nickel‐based catalysts and for the formulation of promising materials for CH_4_ decomposition on the industrial scale. The Ni‐PS catalysts not only produce micrometer‐large and uniform CNTs, but also prevents the typical metal nanoparticle detachment.

Moreover, it showcases the outstanding stability of nickel phyllosilicate under harsh reaction conditions up to 750 °C. Thus, this approach is also of great relevance to the development of active and stable materials applied for thermochemical processes at high temperatures.

## 2 Results and Discussion

### 2.1 Structure and Properties of the Catalysts

**Figure 1:**
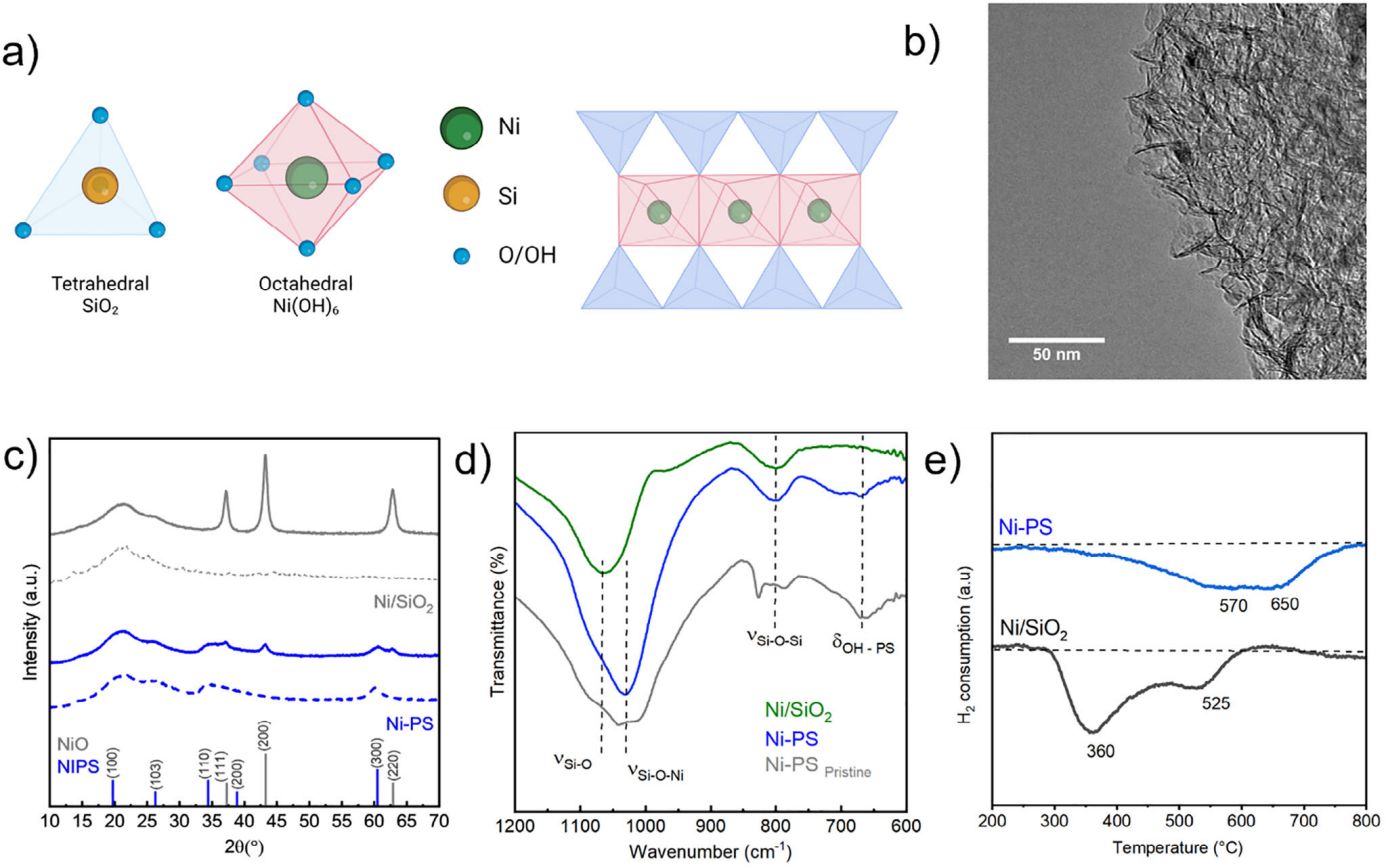
a) schematic representation of the nickel phyllosilicate structure; b) TEM micrograph of calcined Ni‐PS; c) XRD of pristine and calcined Ni‐PS and Ni/SiO_2_; d) FTIR of Ni‐PS and Ni/SiO_2_; e) H_2_‐TPR of Ni‐PS and Ni/SiO_2_.

Metal phyllosilicates are well‐known 2D structures based on transition metals like Ni, Co, or Cu in octahedral coordination with ‐OH and/or O^2−^ ligands. The transition metal can be located between one or two layers of Si in tetrahedral coordination with ‐OH/O^2−^ groups.^[^ ^38^ ^]^ Specifically for nickel, the structure of 2:1 phyllosilicate is schematically represented in **Figure** **1**a, and the structure from DFT is illustrated in Figure S9 (Supporting Information) (ESI).

During the Ni‐PS synthesis (see experimental section), the basic environment promoted by ammonia forms a stable [Ni(NH_3_)_6_]^2+^ complex of intense blue color. After adding the silica source, the highly basic conditions promote the dissolution of Si(OH)_4_ oligomer units. Subsequently, the controlled ammonia evaporation by mild heating promotes the formation of nickel oxyhydroxide species. Thus, condensation and polymerization with dissolved Si(OH)_4_ monomers give rise to the formation of Ni‐PS.^[^ ^39^, ^40^ ^]^ The characteristic lamellar structure of calcined Ni‐PS is observed by TEM in Figure 1b.

Ni‐PS, Ni/SiO_2_, Ni‐PS‐Red, and Ni/SiO_2_‐Red denote the nickel phyllosilicate and state‐of‐the‐art impregnated catalyst after calcination and after reduction, respectively. The crystalline phases of the pristine (dried catalyst before calcination–dotted line) and calcined materials (straight line) were characterized by powder X‐ray diffraction (Figure 1c). Both pristine and calcined Ni‐PS show the characteristic reflections of 2:1 nickel phyllosilicate at 19.5° (100), 26.7° (103), 33.7° (110) and 60.9° (300) (PDF 43–0664). After calcination, the characteristic reflection of the phyllosilicate is preserved, together with some slight diffractions corresponding to NiO. On the contrary, the Ni/SiO_2_ catalyst employed as reference evidence of very sharp and intense reflections at 37.2°, 43.2°, and 62.7°, corresponding to the (111), (200), and (220) reflections of NiO, respectively (PDF 44–1159).^[^ ^22^ ^]^

The nature of the Si─O and ─OH bonds for both Ni‐PS and Ni/SiO_2_ were investigated by FTIR spectroscopy (Figure 1d). The spectra show the characteristic absorbance bands at 1075 and 800 cm^−1^, corresponding to the asymmetric and symmetric stretching of Si─O of the tetrahedral SiO_2_. Nevertheless, Ni‐PS shows clear differences in comparison with Ni/SiO_2_. An additional feature at 667 cm^−1^ is ascribed to the ‐OH bending mode in the 2:1 nickel phyllosilicate structure.^[^ ^27^ ^]^ Meanwhile, the shift from 1075 to 1030 cm^−1^ is attributed to the disappearance of isolated ‐OH groups in the SiO_2_ and the formation of Si─O─Ni bonds.^[^ ^18^, ^40^, ^41^ ^]^ The contributions at 1090, 1007, and 827 cm^−1^ in the pristine Ni‐PS (before calcination) are related to the presence of ammonium nitrate species prior to their removal.^[^ ^42^ ^]^

The reducibility of the catalysts was analyzed by H_2_‐TPR (Figure 1e). The reduction properties of supported metal nanoparticles depend on different properties like the oxidation state, particle size, gas composition and pressure, and metal‐support interaction.^[^ ^43^ ^]^ As can be observed, Ni/SiO_2_ shows a main reduction event at 360 °C, with a second one at 525 °C. In agreement with the reported literature, the first one is attributed to the reduction of bulk NiO particles and the second one is attributed to the reduction of Ni^2+^ ions grafted on the silanol groups on the surface. In the case of Ni‐PS, the strong metal‐support interaction between the layered SiO_2_ and Ni^2+^ in octahedral coordination gives rise to reduction events at higher temperatures, with two maxima at 570 and 650 °C.^[^ ^23^, ^24^, ^44^ ^]^

Both Ni/SiO_2_ and Ni‐PS catalysts are based on 20%wt. Ni. The only distinction lies in their preparation method. This approach enables the incorporation of a high metal loading of Ni^2+^ within the silica structure. This is further verified by SEM studies (Figure S1, Supporting Information). Backscattered electron micrographs clearly show the formation of large particles for Ni/SiO_2_, as well as the high dispersion and absence of agglomerates of the nickel phase for Ni‐PS.

### 2.2 Formation and Stability of Nickel Nanoparticles: In Situ XRD and In Situ TEM Under H_2_

The thermal treatment of certain metal oxides under a controlled atmosphere is an effective method for obtaining supported metal nanoparticles. This route, known as exsolution, is typically applied to structures like perovskites and spinel oxides.^[^ ^45^, ^46^, ^47^, ^48^ ^]^ However, it can also be applied to metal phyllosilicates, obtaining small and well‐dispersed supported nanoparticles. As presented in the section above, nickel phyllosilicate shows strong metal‐support interaction, characteristic of Ni^2+^ cations in octahedral configuration in the layered structure. In this section, in situ X‐ray diffraction together with in situ transmission electron microscopy provide insights into the crystalline nature, stability, and size of supported nickel nanoparticles formed in Ni‐PS‐Red by thermal reduction of Ni‐PS.

**Figure 2:**
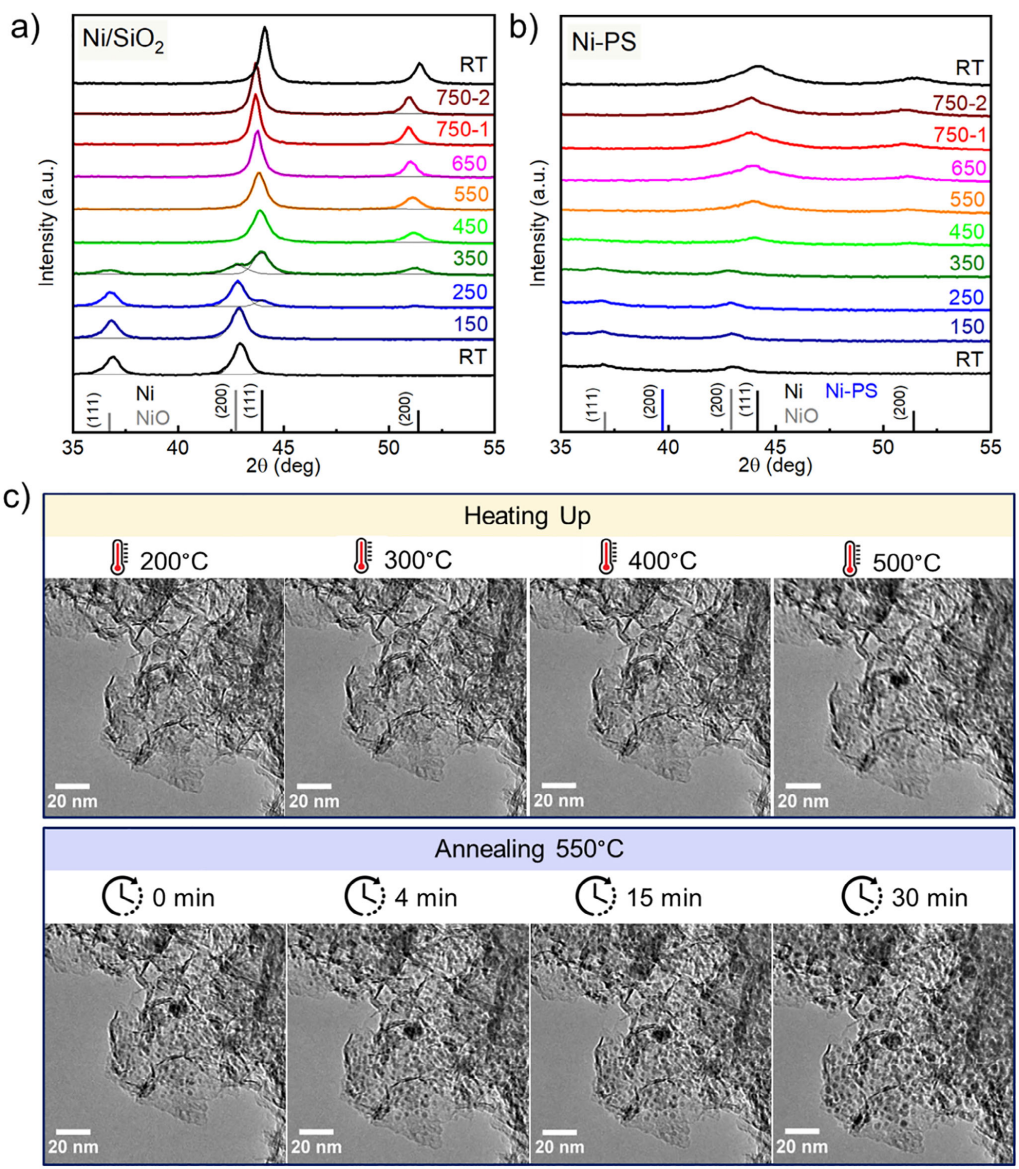
In Situ XRD under hydrogen for a) Ni/SiO_2_ and b) Ni‐PS: each step, i.e. reduction and diffractogram collection, last 1h each. At 750°C two diffractograms were collected; c) In situ TEM of Ni‐PS under reducing conditions.

**Figure** **2**a shows the in situ X‐ray diffractograms for the reference Ni/SiO_2_. Each diffractogram was collected at the referred temperature for 1 h, with a previous reduction step of 1h. Once at 750 °C, the diffractogram was collected twice (i.e., 750–1 and 750–2, respectively). Below 250 °C, the reflections at 37.2° and 43.2° correspond to the (111) and (200) planes of NiO, respectively (PDF 44–1159). At 250 °C, the reduction of NiO is followed by the formation of metallic Ni with reflections at 44° and 51°, corresponding to the (111) and (200) planes, respectively (PDF 04–0850). Under the reduction conditions employed for the study, NiO is fully reduced at 450 °C. The relative ratio of Ni phases versus temperature is shown in Figure S2a (Supporting Information). The crystallite size (calculated applying Scherrer's equation for (111) diffraction planes, shape factor K = 0.9, λ = 1.5406 Å (Cu Kα1)) monotonically increases from 10.2 nm at 450 °C to 17.8 nm at 750 °C (Figure S2b, Supporting Information).

Ni‐PS shows a very different behavior (Figure 2b). The thermal evolution of small and broad reflections suggests nanoparticles of amorphous nature and/or very small size.^[^ ^49^ ^]^ Temperatures above 450 °C are needed for the clear detection of metallic nickel, giving rise to small crystallites of 4.9 nm. Remarkably, at 750 °C the crystallite size slightly increases to 5.9 nm (Figure S2b, Supporting Information). Therefore, very small and well‐dispersed nickel particles of excellent thermal stability can be obtained by employing nickel phyllosilicate as a precursor.

Thermal treatments like reduction or calcination are widely employed for activating the catalyst material. Usually, characterizations are based on ex situ techniques, or in situ bulk techniques like XRD and TPR.^[^ ^19^, ^50^ ^]^ Therefore, it is important to understand the nucleation and growth processes at a local scale, leading to a better understanding of the dynamics of the formation of active sites.^[^ ^51^, ^52^ ^]^ Van den Berg et al. studied the formation of copper nanoparticles employing in situ TEM on a phyllosilicate‐based precatalyst.^[^ ^53^ ^]^ His work derived from a strict experimental protocol allowing the quantification of beam‐induced effects and measures to minimize them. Lately, Turner et al. performed in situ TEM studies on nickel phyllosilicate at 500 °C and 50 mbar of H_2_. Like Van den Berg, a precise experimental protocol was established for quantification of beam‐induced effects during the precatalyst reduction.^[^ ^54^ ^]^ We established a similar protocol adopted to our experimental conditions, where beam‐induced effects are minimized with electron dose below 30 e^−^ A^−2^ s^−1^, reduction temperature of 550 °C, and beam blanked between collections (see experimental details).

Figure 2c shows the TEM results of the thermal evolution of Ni‐PS, showing the characteristic lamellar structure of the phyllosilicate. In agreement with the in situ XRD results, the clear formation of Ni nanoparticles (NiNPs) is not evidenced below 400 °C. At 500 °C, small and dispersed particles start being formed, serving as nuclei for formal NiNPs at 550°C. Once the temperature reached 550 °C, the reduction was followed by 30 min. This temperature was adopted based on H_2_‐TPR results, guaranteeing that there is enough thermal energy transferred to the sample to promote the reduction process and suppress electron beam contributions. As shown in Figure 2d, after only 4 min the layered structure is covered by small and dispersed NiNPs. After 30 min of reduction, it is observed that nickel nanoparticles slightly increased showing a size of 3.6 ± 0.4 nm, in close agreement with the crystallite size observed by in situ XRD.

Like previous findings on Cu‐ and Ni‐based phyllosilicates, nanoparticle formation follows an autocatalytic model. Once the first nuclei are formed, nanoparticle growth describes an autocatalytic process followed by diffusion‐limited growth where it reaches a defined size at the expense of the local Ni^2+^ concentration. Our results agree with Turner et al., leading to the formation of well‐defined and small NiNPs.^[^ ^54^ ^]^ The differences in H_2_ partial pressures suggest that does not significantly affect the particle size in the studied range (50 vs 1.8 mbar in our studies). The strong metal‐support interaction between the NiNPs and the SiO_2_ support prevents the migration and coalescence phenomena that would lead to agglomerates.

### 2.3 Insights into the Reducibility of Ni─O Bonds: a DFT Study

To elucidate the distinct behavior in the reduction of NiO and Ni‐PS from the in situ X‐ray diffractograms and the H_2_‐TPR, a thermodynamic analysis of oxygen (O) reduction in NiO(200) and Ni_3_Si_4_O_10_(OH)_2_(001) surface models were performed using density functional theory (DFT). The 2:1 phyllosilicate structure (Ni_3_Si_4_O_12_H_2_) was modeled as a 3‐layer system, consisting of two Si_2_O_5_ layers (one on top and one on the bottom) and a central Ni_3_(OH)_2_ layer. Additional information on the antiferromagnetic analysis for the NiO model and magnetic convergence for Ni_3_Si_4_O_10_(OH)_2_ are included in Supporting Information (ESI) (Table S1, Figures S5,S6,S7, and S8, Supporting Information). The surface selection was based on surface energy analysis, comparing (200) and (111) facets for NiO, and (001) and (110) facets for Ni_3_Si_4_O_10_(OH)_2_, with details available in ESI (Section 3; Figures S9 and S10, Supporting Information respectively). The discussion here focuses on NiO(200) and Ni_3_Si_4_O_10_(OH)_2_(001) surfaces as they exhibited lower surface energy.

**Figure 3:**
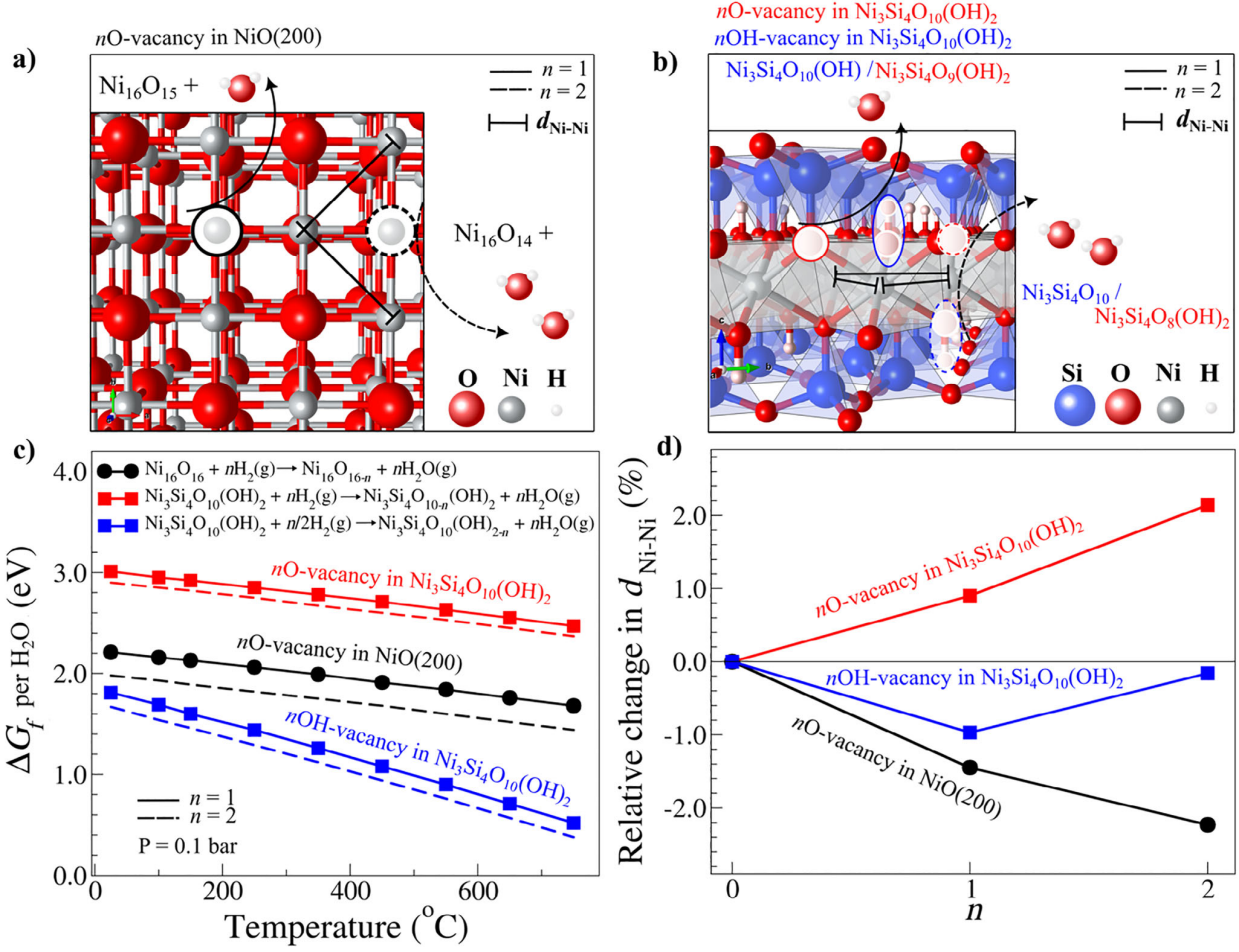
a) Top view of the structure for NiO(200) surface and b) Side view of the structure for Ni_3_Si_4_O_10_(OH)_2_(001) surface. The oxygen vacancies and the Ni atoms used to evaluate changes in the Ni─Ni bond distances are highlighted. c) Gibbs free energy for oxygen vacancy formation (*ΔG*_*f*_) per water (H_2_O) formed as a function of the temperature. *ΔG*_*f*_ is obtained for the oxygen reduction from NiO(200) and Ni_3_Si_4_O_10_(OH)_2_ surfaces with hydrogen (H_2_(g)) to form H_2_O(g) when reducing *n* number of oxygens, with *n* being from 1 to 2. d) Relative changes in the Ni─Ni bond distance (*d*_*Ni* − *Ni*_) as a function of *n*.

**Figure** **3** illustrates the oxygen reduction process for NiO(200) (a) and Ni_3_Si_4_O_10_(OH)_2_(001) (b) when H_2_(g) serves as the reducing agent, leading to H_2_O(g) formation and the generation of an oxygen vacancy. In the NiO(200) model, the formation of the first and second oxygen vacancies (*n* = 1 and 2) involves surface oxygen atoms coordinated with Ni atoms (Figure 3a). For the Ni_3_Si_4_O_10_(OH)_2_(001) model, two distinct types of oxygen vacancies were studied: one corresponding to the reduction of an OH group bonded to Ni atoms (indicated by blue ovals), and another involving the reduction of O atoms coordinated with both Si and Ni atoms (indicated by red circles) in Figure 3b.

The thermodynamic analysis reported in Figure 3c is based on the calculation of the Gibbs free energy (Δ*G_f_*) for the formation of the oxygen vacancies per H_2_O(g) produced. Three cases were explored, which are represented by the following chemical reactions and used to obtain Δ*G_f_*:

(1) $$Ni_{16}O_{16}+nH_{2}\left(g\right)\to Ni_{16}O_{16-n}+nH_{2}O\left(g\right)$$

(2) $$Ni_{3}Si_{4}O_{10}{\left(OH\right)}_{2}+\frac{n}{2}H_{2}\left(g\right)\;\to Ni_{3}Si_{4}O_{10}{\left(OH\right)}_{2-n}+nH_{2}O\left(g\right)$$

(3) $$Ni_{3}Si_{4}O_{10}{\left(OH\right)}_{2}+nH_{2}\left(g\right)\;\to Ni_{3}Si_{4}O_{10-n}{\left(OH\right)}_{2}+nH_{2}O\left(g\right)$$

Then,

(4) $$\Delta \;G_{f}=\Delta H_{f}\;-T\Delta \;S_{f}=\Delta E_{f}+\Delta E_{f}^{ZPE}+\Delta C_{f}dT-T\Delta S_{f}$$

(5) $$\Delta E_{f}=\Sigma \;E_{tot}^{product}-\Sigma E_{tot}^{reactacts}$$

where Δ*H_f_* refers to the change in the enthalpy of the process, Δ*E_f_* represents the change in energy, $\Delta E_{f}^{ZPE}$ denotes the change in the zero‐point energy, Δ*C_f_dT* indicates the change in the heat capacities, Δ*S_f_* represents the change in the entropy, and *T* is the temperature. Since $\Delta E_{f}^{ZPE}$, Δ*C_f_dT*, and *T*Δ*S_f_* depend on H_2_O(g) and H_2_(g), they are calculated at different temperatures and 0.1 bar, emulating the H_2_ partial pressure during the catalyst reduction, and within the ideal gas limit as implemented in the thermochemistry module available in the atomistic simulation environment package.^[^ ^55^ ^]^ Supplementary data can be found in Section 4 from the ESI (Tables S2 and S3, Supporting Information). Therefore, the Δ*G_f_* can be analyzed as a function of the temperature (Figure 3c).

As expected, an increase in the temperature favors oxygen reduction for all cases, whether forming one (symbols connected by solid lines) or two (dashed lines) oxygen vacancies. Interestingly, the formation of two oxygen vacancies per H_2_O(g) is ≈0.2 eV more favored than the formation of a single oxygen vacancy in NiO(200), while this difference is less than 0.2 eV for both types of vacancies studied in Ni_3_Si_4_O_10_(OH)_2_(001). This indicates that the formation of oxygen vacancies becomes increasingly favorable as the number of vacancies increases, with a more pronounced effect observed in NiO(200) compared to Ni_3_Si_4_O_10_(OH)_2_(001).

The comparison of Δ*G_f_* between the three cases shows that vacancy formation in Ni_3_Si_4_O_10_(OH)_2_(001) through OH group reduction (blue) is thermodynamically more favored than in NiO(200). However, vacancy formation is more favorable in NiO(200) than Ni_3_Si_4_O_10_(OH)_2_(001) reduced through O bonded to Si and Ni atoms (red). This suggests that OH reduction in Ni_3_Si_4_O_10_(OH)_2_(001) is significantly easier than its O reduction. If both OH and O vacancies are involved in Ni_3_Si_4_O_10_(OH)_2_(001) reduction, reducing O bonded to Si and Ni could limit the process unless a significant kinetic barrier affects OH reduction. In addition, it should be considered that the ratio between OH and O entities is 1:5, respectively. Consequently, the reduction of Ni_3_Si_4_O_10_(OH)_2_(001) would be less favorable than that of NiO(200), requiring even higher temperatures. These results are consistent with our experimental evidence.

Figure 3d shows the relative change in the Ni─Ni distance (*d*_*Ni* − *Ni*_) as a function of the number of vacancies for each case. After the formation of a single vacancy in both models, the Ni─Ni distance changes by ≈|1.0|%. Previous studies calculating Ni clusters with 13, 55, and 147 atoms suggest that the average *d*_*Ni* − *Ni*_ in a Ni_147_ is ≈2.44 Å.^[^ ^56^ ^]^Compared with the *d*_*Ni* − *Ni*_ in NiO(200) (2.94 Å), this represents a contraction of ≈17.0% in the Ni─Ni distance when a cluster of Ni_147_ is formed. This suggests that the NiNPs formation may involve the creation of more than one O vacancy, motivating the study of a second vacancy in this study.

The formation of a second vacancy led to a contractive effect of up to 2.0% in the Ni‐Ni distance for atoms around the vacancies in NiO(200), while an expansive effect of up to 2.0% is observed for O vacancies formation in Ni_3_Si_4_O_10_(OH)_2_(001). For OH vacancies formation in Ni_3_Si_4_O_10_(OH)_2_(001), a smaller contractive effect (≈0.9%) is observed compared to NiO(200) when a single vacancy is generated, and a negligible effect (0.16%) is seen when two OH vacancies are involved. These findings indicate that structural changes in Ni atoms differ significantly depending on the material and the type of vacancy. Given that oxygen reduction drives the formation of NiNPs, further mechanistic studies focused on these structural changes could provide valuable insights into the processes governing metal nanoparticle formation.

### 2.4 Catalytic Methane Decomposition: Switching from Tip to Base Growth of Carbon Nanotubes

In general terms, carbon nanostructure formation can be described as a four‐step process: a) hydrocarbon C─H bond dissociation at the nickel surface, b) carbon diffusion through the bulk and/or the surface of the metal site, c) carbon precipitation from the edges once the metal nanoparticles reach saturation and d) Ni diffusion causing step restructuring. Independently of the metal‐support interaction, CNTs formation and their morphologies are the result of the balance between C‐H activation, carbon diffusion, and precipitation rates leading to C─C coupling and Ni diffusion.^[^ ^57^ ^]^ In strict terms, carbon nanotubes (CNTs) consist of concentrically aligned graphene layers forming parallel tubular structures, whereas carbon whiskers refer to graphene layers inclined relative to the fiber axis. For simplicity, CNTs are defined broadly to include any graphitic nanofiber, whether the graphene layers are aligned or inclined relative to the fiber axis.

Methane decomposition is a structure‐sensitive reaction, where the particle size has a strong influence on the carbon yields, deactivation rates, and structure of the deposited carbon. F. Abil Pedersen et al. investigated CH_4_ dissociation on a Ni(141313) single crystal, a Ni(111) surface with a low density of steps.^[^ ^58^ ^]^ The combination of experimental results and DFT calculations on Ni(111) and Ni(211) allow for the comparison of CH_4_ dissociation on flat terraces and step edges, respectively. The activation energy on Ni(211) steps is 17 kJ mol^−1^ lower than in flat (111) surfaces, in agreement with the enhanced stability of •CH_X_ intermediates. The intrinsic sticking probabilities, a measure of the likelihood of CH_4_ adhering to the surface, were found to be 2.8 × 10^−7^ for steps and 2.1 × 10^−9^ for terraces at 500 K. This difference aligns with the calculated activation energy difference.

Later, experimental and theoretical studies on model nanoparticles composed of Ni(111) and Ni(100) facets demonstrated that high‐index planes promote CH_4_ dissociation and carbon surface diffusion before CNTs formation. More specifically, B_5_ sites are specific active sites on the steps of nanoparticle surfaces. These B_5_ sites are structurally unique, typically associated with a configuration where five metal atoms create a highly reactive local environment. It was found that these sites not only enhance CH_4_ dissociation compared to flat terraces but also increase the stability of •CH_3_ intermediate.^[^ ^59^, ^60^ ^]^

Nevertheless, prior to these findings, it was already established that the decomposition barrier of CH_4_ is lower than the energy required for graphene growth. DFT calculations and in situ TEM proved that carbon binds strongly at step‐edge sites on Ni(211) than planar Ni(111) facets, suggesting that nucleation of graphene layers is preferred on these defective sites driving the growth of CNTs.^[^ ^32^, ^34^ ^]^ Whether carbon diffusion occurs on the surface or within the bulk of the nanoparticles, is discussed in the following section.

Specifically for Ni‐SiO_2_ catalysts, Xu et al. reported that at 650 °C particles below 10 nm were not active toward CNTs growth due to carbon encapsulation, meanwhile particles larger than 20 nm present high activity.^[^ ^61^ ^]^Takenawa et al. observed that at 550 °C, only particles between 40–100 nm produced a larger amount of carbon whiskers.^[^ ^62^ ^]^ Similarly, Chen et al. observed an optimum at 38 nm performing the reaction at 580 °C.^[^ ^63^ ^]^ The authors claimed that smaller particles suffer from deactivation due to carbon encapsulation, while larger ones lead to poor CH_4_ decomposition due to low surface area. Similarly, Liang et al. observed that at 800 °C particles below 14 nm were inactive due to encapsulation, while particles of 25 nm were active.^[^ ^64^ ^]^ Pinilla et al. observed that between 500 and 700 °C the optimum size of Ni domain lies between 10–30 nm, with poor activity for crystallites larger than 50 nm.^[^ ^65^ ^]^

**Figure 4:**
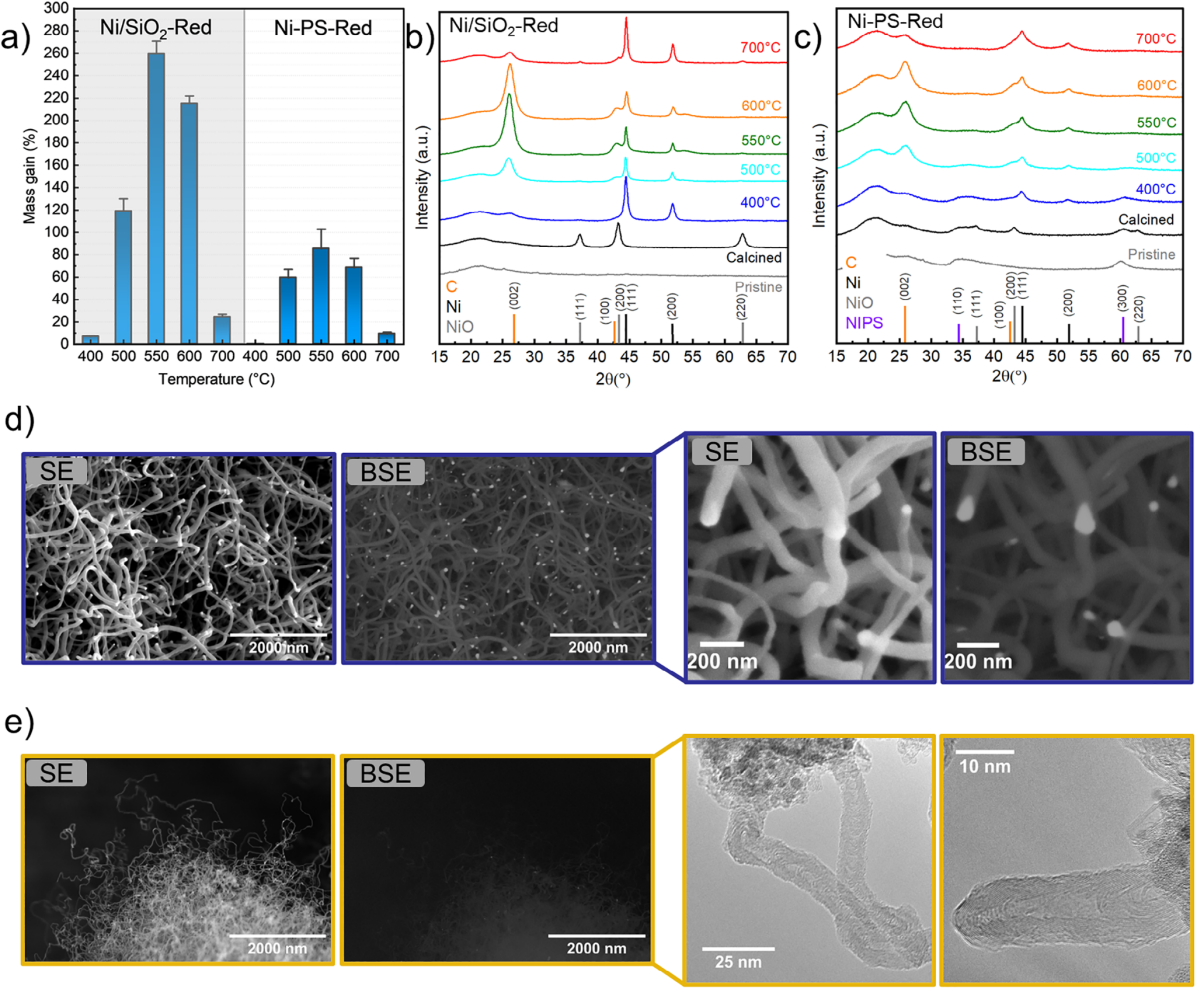
a) Mass gain for Ni/SiO_2‐_Red and Ni‐PS‐Red (mean ± SD value, N = 3); XRD of spent samples: b) Ni/SiO_2_‐Red and c) Ni‐PS‐Red; d) SEM of spent Ni/SiO_2_‐Red; e) SEM and HR‐TEM of spent Ni‐PS‐Red.

**Figure** **4**a shows the mass gain of the samples, relative to the initial mass at different temperatures, attributed to CNTs formation.^[^ ^66^, ^67^ ^]^ At 550 °C, Ni/SiO_2_‐Red and Ni‐PS‐Red showed a maximum of 259 ± 10% and 85 ± 16%, respectively. This agrees with reported results, which show a maximum for Ni‐silica‐based catalysts between 500–600 °C.^[^ ^65^, ^66^, ^68^ ^]^ Due to the endothermic nature of CH_4_ decomposition, below 550 °C the reaction is thermally limited. In agreement, at 400 °C a small mass gain of 7 ± 1% was observed for Ni/SiO_2_‐Red. No activity was observed for Ni‐PS‐Red.

Temperatures above 650 °C lead to conversions above 78% in thermodynamic equilibrium.^[^ ^69^ ^]^ However, particle size changes caused by sintering, and their impact on catalytic activity, must also be considered. Consistently, a decline in activity was observed at temperatures above 550 °C. At 700 °C, Ni/SiO_2_‐Red and Ni‐PS‐Red showed a mass gain of 24 ± 4% and 9 ± 2%, respectively.

Figure 4b shows the XRD diffractograms of spent Ni/SiO_2_‐Red. At 400 °C, a slight diffraction peak corresponding to the (002) plane of graphitic carbon is present. The Ni crystallite size is estimated at 13 nm (for calculations, see experimental details). At 550 °C, the graphitic peak is dominating, in agreement with the high catalytic activity. Meanwhile, at 700 °C the formation of graphitic carbon is low, with a Ni crystallite size of 17 nm.

Similarly, Figure 4c shows the XRD diffractograms of spent Ni‐PS‐Red. At 400 °C, no diffraction peak corresponding to carbon is observed. At 550 °C, a dominant graphitic peak appears, consistent with the higher catalytic activity reported in Figure 4a. At 700 °C the formation of graphitic carbon is notably decreased.

**Figure 5:**
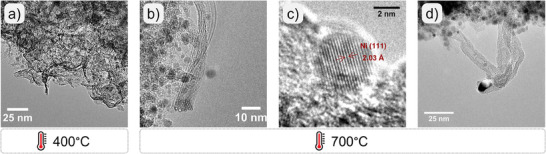
TEM micrographs of Ni‐PS‐Red after reaction at a) 400 °C and b–d) 700 °C.

**Figure** **5**a shows the TEM micrograph of Ni‐PS‐Red tested at 400 °C. The presence of small and dispersed Ni nanoparticles and the absence of CNTs formation verifies that CH_4_ decomposition did not take place. Meanwhile, the micrograph of Ni‐PS‐Red tested at 700 °C clearly shows the formation of different CNTs structures (Figure 5b–d). Surprisingly, NiNPs do not show significant sintering, with particles below 5 nm. In agreement with the ex situ XRD, the distance between parallel lattice fringes in Figure 4c corresponds to metallic Ni (2.03 Å for Ni(111) vs 2.12 Å for NiO(200)). It is inferred that carbon encapsulation preserved the nanoparticles from further oxidation and deactivated the catalyst. Some isolated bundles of very thin CNTs with diameters as small as 0.68 ± 0.09 nm were observed (Figure 4b). Meanwhile, some isolated larger particles above 10 nm led to the formation of multiwalled CNTs, with detachment of the metal nanoparticles from the support (Figure 4d).

In agreement with previous studies, 550 °C is the optimal temperature for Ni‐SiO_2_ catalysts, leading to a balance between thermal activation of CH_4_, carbon and nickel diffusion, and precipitation rates. Figure 4d,e shows the scanning and transmission electron micrographs of the spent catalysts at 550 °C. Ni/SiO_2_‐Red formed CNTs of broad diameters (81 ± 16 nm; Figure 4d upper case in blue frame) in comparison with Ni‐PS‐Red (16 ± 3 nm; Figure 4e lower case in yellow frame), as also shown in Figure S4 (Supporting Information).

In scanning electron microscopy, backscattered electrons (BSE) are more likely to be emitted from atoms with higher atomic weights, creating brighter regions in the image for heavier elements. This atomic number contrast allows for easy differentiation between materials of different atomic compositions like nickel and carbon. The backscattered electron micrograph of spent Ni/SiO_2_‐Red evidence the typical tip‐growth mechanism with Ni particles lifted from the support. In this process, carbon adsorbs at the nanoparticle front surface and precipitates at the bottom of the metal particle in a concerted motion with Ni atoms being expelled from the C‐Ni interface to diffuse to the Ni front surface. Under reaction conditions, their morphology drastically changes forming elongated conical structures, as observed in the high magnification SEM micrographs. Another characteristic of the tip‐growth mechanism is the formation of CNTs of similar diameters to nickel nanoparticles.^[^ ^32^, ^70^ ^]^

**Figure 6:**
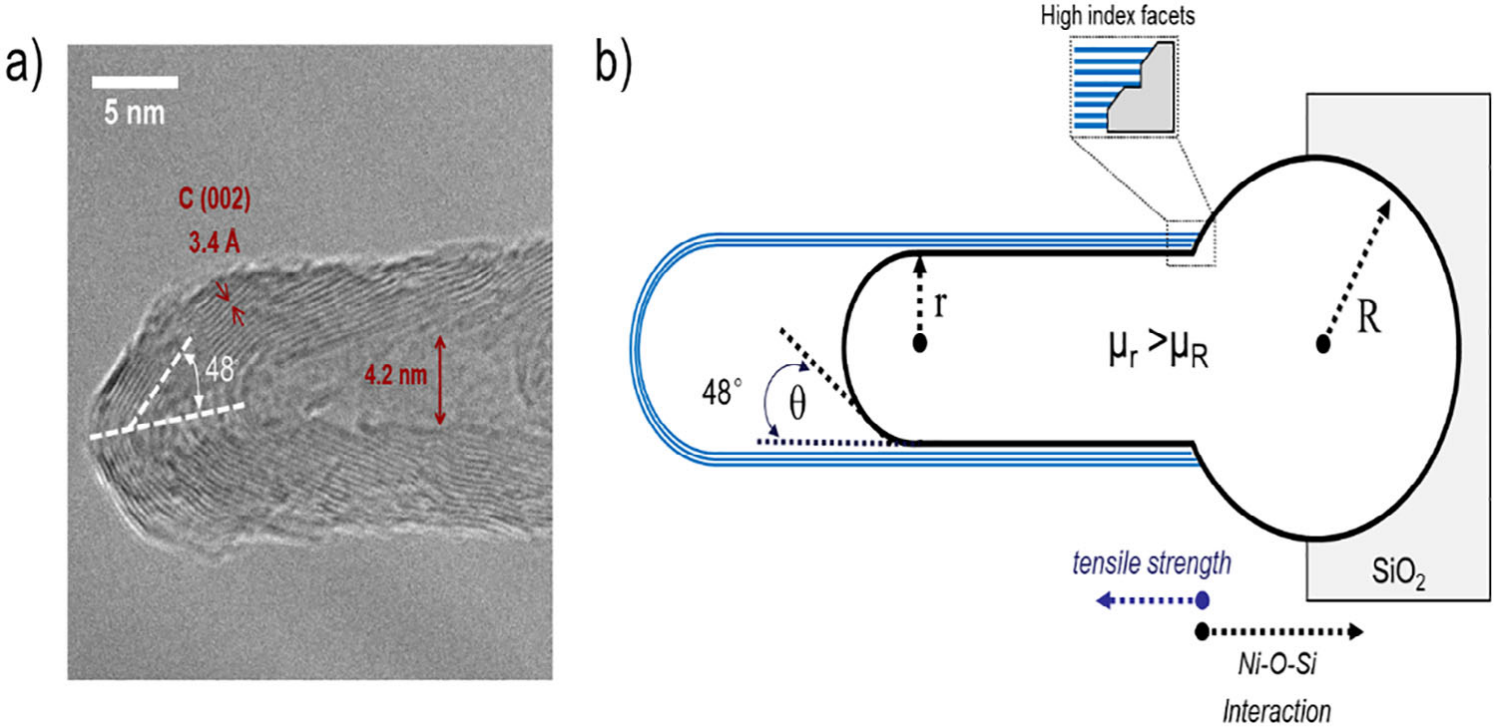
a) HR‐TEM of a single carbon nanotube grown in the nickel phyllosilicate showing a base growth mechanism; b) Schematic representation of the dynamics during carbon nanotube growth. Adapted from ^75^ with permission from the American Chemical Society to describe the base‐growth mechanism observed in this work.

Remarkably, the mechanism of CNTs growth in Ni‐PS‐Red is completely different. As observed in Figure 4e, backscattered electrons SEM shows no evidence of detached Ni nanoparticles on the tips of the CNTs. Thus, suggesting that metal sites remain attached to the support. This is further verified by TEM, where Ni particles remained on the support forming CNTs following a base growth mechanism. **Figures** **6**a and S7 (Supporting Information) show in greater detail the carbon structures formed by Ni‐PS‐Red. CNTs lattice fringes show a value of 0.34 nm, in agreement with the (002) plane of graphitic C. The hollow structures possess a diameter of 4.2 nm, in close agreement with the size of the nickel nanoparticles. In addition, all carbon nanotubes show a close‐end structure, characteristic of the base‐growth mechanism. Interestingly, and unlike the typical tip‐growth, graphene layers at the tip of the multiwalled CNTs are not oriented in a parallel direction to the hollowed structure but stacked with an angle of 48 ± 2 degrees with respect to the growth direction. Therefore, unlike the tip‐growth process, it is inferred that after CH_4_ cracking and carbon diffusion the strong metal‐support interaction drives the carbon precipitation at the apex of the nanoparticle instead of the bottom.^[^ ^10^, ^71^, ^72^ ^]^ Thus, CH_4_ molecules decompose at the interface between the active sites and the support, since CH_4_ cannot diffuse at the top of the closed‐ended CNTs.

Helveg et al. used in situ transmission electron microscopy at 1 mbar of CH_4_ to study the dynamic evolution of nickel particles supported on MgO.^[^ ^32^ ^]^ Under these conditions, the smaller Ni particles between 5 and 20 nm adopt an elongated shape, forming tubular carbon structures with graphene layers aligned parallel to the fiber axis. In contrast, particles larger than 20 nm develop a pear‐like shape, resulting in graphitic nanofiber structures where the graphene layers are inclined relative to the fiber axis. In both cases, metal nanoparticles are lifted from the support following a tip‐grown mechanism. Theoretical calculations highlight the critical role of step edges on Ni nanoparticles, where carbon atoms preferentially bind, promoting graphene layer formation. Carbon diffuses across the Ni surface rather than through the bulk, while Ni atoms migrate away from the graphene–Ni interface. This dynamic surface diffusion aligns with the observed reshaping of Ni particles during growth.

The dynamic reshaping of metal nanoparticles significantly impacts the final structure and properties of CNTs. Moseler et al. combined molecular dynamic simulations with in situ TEM experiments to describe the elastic nature of the morphological evolution of nickel nanoparticles during hydrocarbon cracking and CNTs formation.^[^ ^73^ ^]^ The system can be modeled as two hemispherical surfaces of different radii, interconnected by a metallic tail. The differences in chemical potential between the two surfaces act as a driving force for the metal nanoparticle elongation and reshaping due to capillary‐driven surface diffusion of nickel atoms on a crystalline core. Both the intra‐tube metallic tail and the larger hemispherical surface consist of crystalline planes of low‐index surfaces. However, the interface between these structures comprises high‐index surface kinks and steps, where carbon precipitation and nanotube formation take place. The surface diffusion of nickel atoms from the tail to the head of the nanoparticle through the metastable high‐index surfaces is considered the limiting step of the process.

Based on our experimental evidence observed by SEM and HR‐TEM, carbon nanotube growth follows a similar phenomenon on both Ni/SiO_2_‐Red and Ni‐PS‐Red. However, for Ni‐PS‐Red, the elongation process occurs in the opposite direction, with the head of the metal nanoparticle remaining attached to the support, and its elongation occurring tangentially. The interaction between the nickel nanoparticle and the silica support is stronger than the tensile force applied during CNT growth, thus preventing the detachment of the active sites. A scheme of the process is represented in Figure 6b. Ni/SiO_2_‐Red follows the typical tip‐growth mechanism with detachment of the metal sites.

Regarding the higher carbon yields of Ni/SiO_2_‐Red, it is inferred that it is promoted by a) the formation of larger and more active nickel particles and b) the favored CH_4_ diffusion and access to the nickel particles following a tip‐growth mechanism relative to base growth. This agrees with the crystallite domains observed in the XRD diffractograms, and the diameters of the CNTs and Ni nanoparticles observed by SEM and HR‐TEM of the spent samples. Remarkably, and in disagreement with the literature so far, our findings reveal that Ni nanoparticles below 5 nm can also activate CH_4_ decomposition forming micrometer‐long multiwalled CNTs under atmospheric partial pressures.^[^ ^27^, ^61^, ^63^, ^64^, ^68^ ^]^ This is attributed to the strong metal‐support interaction of the phyllosilicate that prevents the detachment and encapsulation of the metal particles. For both Ni/SiO_2_‐Red and Ni‐PS‐Red, temperatures above 600 °C led to a higher CH_4_ cracking rate than carbon diffusion and precipitation. Thus, leading to encapsulation and catalysts deactivation.^[^ ^71^, ^74^ ^]^

Despite the lower catalytic activity, nickel phyllosilicate as a catalyst precursor leads to stable and active metal nanoparticles that remain anchored to the support. The preparation method is easily scalable and highly reproducible, with metal loadings as high as 20%, and the diameter of the produced carbon nanotubes is highly uniform. From an industrial point of view, this is especially attractive for preparing supported catalysts that could be regenerated without loss of the active sites, leading to highly valued CNTs and CO_2_‐free H_2_ production.

### 2.5 Insights Into the Active Phase: In Situ XRD Under CH_4_ Decomposition

Different reaction mechanisms and controlling steps have been proposed for the CH_4_ decomposition on supported nickel catalysts. Surprisingly, there are still reports that debate whether carbon diffuses through the bulk or at the surface.^[^ ^14^, ^30^, ^31^, ^75^ ^]^ Several studies claimed that NiC_3_ species are in a stable intermediate phase during CNTs formation.^[^ ^36^, ^37^, ^76^ ^]^ Nevertheless, these studies are based on post‐mortem characterizations. Thus, NiC_3_ phases can be formed after cooling down the samples, and it does not correspond to the dynamic conditions under reaction atmospheres.

The first in situ TEM describing CNTs growth by CH_4_ decomposition was reported by Helveg et al., using a commercial Ni/MgAlO_4_ catalyst.^[^ ^32^ ^]^ This study proved that nickel nanoparticles suffered from reshaping under reaction conditions, without the formation of nickel carbide species. Supported by theoretical calculations, carbon and nickel atoms diffuse on the surface instead of the bulk. Thus, no driving force is present for carbon incorporation within the octahedral bulk structure of Ni.^[^ ^34^ ^]^

Moreover, it was postulated that the tip growth mechanism of CNTs is limited by the surface diffusion of the carbon species. Similarly, NiC_3_ phases were not evidenced by in situ XRD onto Ni(170nm)/SiO_2_ thin films. Correlated with in situ XPS, it is postulated that a Ni‐C solid solution is formed within the first atomic layers.^[^ ^35^ ^]^ Similarly, Hofmann et al. did not detect the formation of NiC_3_ using time‐resolved XPS.^[^ ^77^ ^]^However, these studies were based on hydrocarbon decomposition on thin films instead of discrete metal nanoparticles and the use of C_2_H_2_ instead of CH_4_. Thus, carbon solubility and diffusion rates due to side effects and the nature of the carbon source were not contemplated.

In contrast, Chen et al. analyzed the effect of CH_4_ dissociation over Ni films at 423 °C. Employing near ambient pressure XPS, a shift toward lower C1s binding energies was attributed to the formation of Ni_3_C species.^[^ ^78^ ^]^ Recently, using synchrotron‐based in situ XRD, Gili et al. reported the formation of NiC_X_ species due to CH_4_ decomposition at 500 °C on Ni/MgO. Under reaction conditions and after CH_4_ dissociation, the fcc lattice of metallic nickel expands reaching a maximum corresponding to the NiC_0.008_ composition followed by its contraction to metallic nickel due to carbon precipitation.^[^ ^79^ ^]^ The carbon atomic concentration corresponding to 0.82% surpasses the reported solubility of 0.22% for carbon in Ni films at 500 °C. This is attributed to the increased C solubility due to nanometric size effects.^[^ ^61^ ^]^

**Figure 7:**
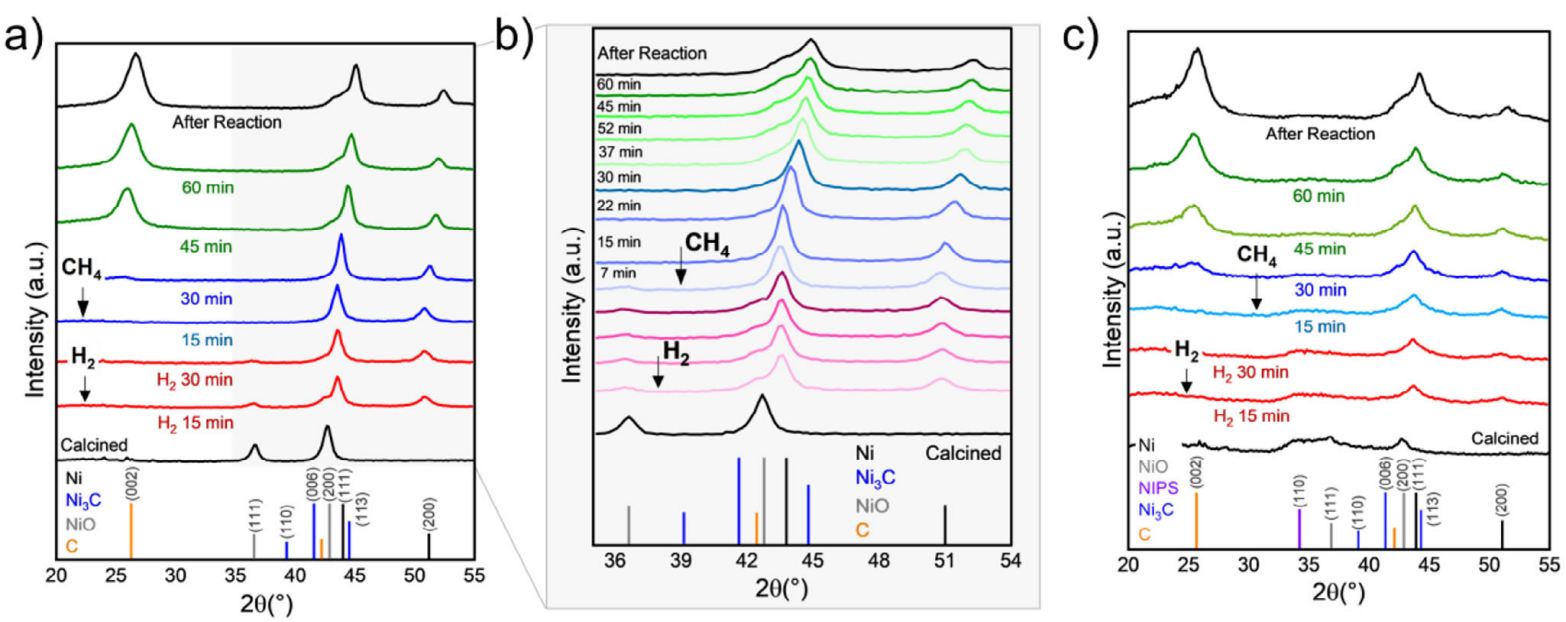
In situ XRD under reduction and methane decomposition conditions at 550° C: a, b) Ni/SiO_2_; c) Ni‐PS.

In situ XRD experiments allow a clear comprehension of the dynamics of the real active phase. All experimental conditions (i.e., temperature, gas composition, GHSV, and reaction time) were kept identical as in the catalytic tests. **Figure** **7**a shows the evolution of Ni/SiO_2_ catalyst. As can be observed, NiO is reduced to Ni during the reduction pretreatment. Once CH_4_ flows, the characteristic (002) plane of graphitic carbon appears after 30 min, and the reflections intensify after 45 min. Figure 7b shows a detailed study within 2θ (35–54°), where there is no evidence of NiC_3_ formation under the reaction conditions applied in this study. The gradual shift toward higher 2θ values during CH_4_ flow is related to the lifting up of the catalyst in the sample holder due to CNTs formation.

Similar conclusions can be drawn from the in situ XRD analysis of Ni‐PS, which shows no evidence of Ni_3_C species (Figure 7c). We acknowledge that our study provides information on the bulk state of nickel under dynamic conditions, without information on the catalytically active surface layer. However, it provides solid evidence that after CH_4_ cracking, there is no carbon diffusion through the bulk of the nickel particles and discarding Ni_3_C as a stable phase during CNTs growth.

## 3 Conclusion

Nickel phyllosilicate is an exceptional catalyst precursor forming small nanoparticles below 5 nm, which remain highly stable even under harsh reducing conditions up to 750 °C.

Experimental evidence indicates that the strong metal‐support interaction between the nickel and the support inhibits particle sintering and detachment during methane decomposition. In addition, a theoretical model of 2:1 nickel phyllosilicate has been calculated using DFT to provide insights into the reducibility of Ni─O bonds, revealing that the high temperatures required for reduction are related to the reduction of the apical oxygen, which is bonded to silicon in the layered precursor (O atoms within the Ni─O─Si bonds).

At 550° C, CH₄ cracking occurs with an optimal balance among carbon dissociation, diffusion, and precipitation rates, resulting in the production of highly uniform carbon nanotubes. Notably, the growth mechanism transitions from the conventional tip‐growth to a base‐growth mechanism, which is associated with a stronger metal‐support interaction (as verified by H_2_‐TPR, in situ TEM, and in situ XRD) compared to the tensile forces exerted during carbon nanotube growth. Consequently, the nickel nanoparticles stay anchored to the support, enabling the formation of carbon nanotubes with closed tip‐ends.

In contrast, the Ni/SiO_2_ state‐of‐the‐art catalysts prepared by impregnation promote the growth of ticker CNTs with detached Ni particles following a tip‐growth mode. This is related to the weakly anchored Ni particles that lead to agglomeration and detachment from the SiO_2_ support.

This study provides the first evidence that nickel particles smaller than 5 nm can decompose CH₄ at atmospheric pressure conditions to produce micrometer‐large, multi‐walled carbon nanotubes with exceptionally narrow diameters of 16 ± 3 nm, rather than encapsulating carbon leading to deactivated nickel sites. Additionally, it has been confirmed that NiC_3_ species do not form during CH₄ decomposition within the bulk phase of the particles.

Overall, this work offers valuable insights for designing active and stable supported nanoparticles for methane cracking, positioning nickel phyllosilicate as a promising candidate to address the key challenges of this process toward large production of highly added value carbon nanotubes with emerging applications in key societal needs.

## 4 Experimental Section

### Chemicals

All chemicals were used as received without any further purification. Silica Davisil was obtained from Sigma‐Aldrich, Germany. Nickel nitrate hexahydrate (99%) was purchased from Sigma–Aldrich, Germany. Ammonium hydroxide (28%) was obtained from Carl Roth, Germany. Water HPLC grade was obtained from Honeywell Riedel‐de‐Haën, Germany.

### Synthesis of Nickel Phyllosilicate (Ni‐PS)

The catalysts were prepared by a modified ammonia evaporation method. In a typical protocol, 3.6 g of nickel nitrate was dissolved in 45 ml of ammonium hydroxide solution in a 100 mL round flask. The nominal metal loading was fixed at 20% wt. After vigorous stirring for 45 min, an intense blue solution was formed, characteristic of the formation of the [Ni(NH_3_)_6_]^2+^ complex. Afterward, 3 g of SiO_2_ was added. The round flask was sealed, and the suspension was stirred overnight at room temperature. As a final step, the lid was removed, and the suspension was heated at 70 °C for 4h to promote ammonia evaporation and phyllosilicate formation. This could be visualized with the evolution of the suspension from light blue to green turquoise.

The green turquoise solid was dried overnight in static air at 100 °C and calcined at 550 °C applying a heating rate of 10 K min^−1^ and a dwell of 4 h, under 300 ml min^−1^ flow of 21% O_2_/N_2_. The calcined catalyst precursor was named Ni‐PS.

### Synthesis of Reference Catalyst (Ni/SiO_2_)

The catalysts were prepared by incipient wetness impregnation. In a typical protocol, 3 g of the commercial silica Davisil® support was impregnated with 2.4 ml of nickel nitrate solution containing the desired amount of metallic precursor. The nominal metal loading was fixed at 20% wt. The catalyst was dried at 100 °C overnight and calcined at 550 °C for 4 h applying a heating rate of 10 K min^−1^ and a 300 mL min^−1^ flow of 21% O_2_/N_2_. The calcined catalyst precursor was named Ni/SiO_2_.

### Catalytic Methane Decomposition

The catalytic experiments were carried out at atmospheric pressure and at temperatures between 400 and 700 °C. 200 mg of sample was loaded on a ceramic crucible and placed inside a quartz tube with an inner diameter of 35mm. The quartz tube was then placed on a horizontal furnace electrically heated. In an activation step, the catalysts were first heated to 550 °C with 10 K min^−1^ and reduced in situ for 30 min in 200 mL min^−1^ of 10% H_2_/He. The activated catalysts after reduction were named Ni‐PS‐Red and Ni/SiO_2_‐Red, respectively.

Afterward, the H_2_ flow was switched off and 75 mL min^−1^ CH_4_ was fed, giving rise to 30% CH_4_ in He. The GHSV corresponds to 76.5 l h^−1^ g^−1^. As an indicator of the catalytic activity, and in agreement with the reported literature, the carbon yield was calculated as 100 • (m_f_ – m_0_) / m_0_; where m_f_ is the final mass and m_0_ is the initial mass, respectively.^[^ ^66^, ^67^ ^]^

### Characterization

The morphology and size distribution of the nanoparticles were analyzed by Transmission Electron Microscopy (TEM) in a FEI Tecnai T20 microscope operating at 200 kV, using a FEI single tilt holder. Samples were prepared by direct contact with the powder catalysts on lacey carbon 400‐mesh gold grids (Agar scientific).

In situ TEM experiments were performed in a FEI Titan ETEM operating a 300 kV. The differential pumping system was employed for the dosing of reacting gases into the microscope column. For the sample preparation, a few milligrams of the specimen were dispersed in 2 mL of absolute ethanol and homogenized to have a stable suspension. One microliter of the suspension was drop‐casted on the center of the wildfire microchip (Denssolutions, previously cleaned with plasma) and left to room temperature for drying.

To ensure that the TEM observations represent the processes intrinsic to the reduction, a beam‐insensitive approach was applied as follows, considering previous works and adapted to our conditions of CH_4_ decomposition in the reactor:^[^ ^53^, ^54^ ^]^

Prior to any experiment, the sample was kept in the microscope collum overnight. The micrographs were taken at temperatures above 200 °C, avoiding any beam‐induced changes to the morphology of the layered structures, which remained stable in the range of study (200–550 °C). A reduction temperature of 550 °C and a heating rate of 10 °C min^−1^ was selected because the thermal energy supplied to the sample was sufficient to thermally promote nucleation and growth rates, surpassing those driven by the electron beam. This temperature was also chosen for the reduction in the reactor prior to CH_4_ decomposition. The total pressure in the column was 1.8 mbar H_2_, and the electron dose rate was kept below 30 e Å^−2^ s^−1^ to avoid beam‐induced effects. A series of micrographs were acquired with an acquisition of 2 frames per second and the beam was blanked between exposures.

Scanning electron microscopy studies (SEM) were performed on a Helios 5 Hydra Dual Beam (ThermoFisher Scientific) operating a 5kV and 0.2 nA. Samples were prepared by direct contact with the powder catalysts on lacey carbon 400‐mesh gold grids (Agar scientific).

Ex situ X‐ray diffractograms were acquired in Bragg–Brentano geometry on a PANalytical Empyrean with Cu Kα radiation (λ = 1.54 Å) over an angular range from 10° to 90° and a scan rate of 1.3° min^−1^. The crystallite size (calculated applying Scherrer's equation for (111) diffraction plane, shape factor K = 0.9, λ = 1.5406 Å (Cu Kα1))

In situ X‐ray diffraction measurements were performed using a PAN‐Analytical X'PERT PRO diffractometer equipped with an Anton Paar XRK‐900 furnace, using Ni filtered Cu Kα radiation (λ = 1.54 Å). The furnace was connected to a gas handling system able to provide He, N_2_, H_2,_ and CH_4_ controlled by their respective mass flow controllers. All conditions were adopted to maintain the same GHSV to 76.5 l h^−1^ g^−1^.

For in situ reduction experiments, 100 mg of sample was loaded and heated at a heating rate of 10 K min^−1^ under 100 mL min^−1^ flow of 10% H_2_ in He. XRD diffractograms were collected from 10° to 90° and a scan rate of 1.3° min^−1^ at different temperature steps and at room temperature before and after each experiment. Each step, i.e. reduction and diffractogram collection, last 1h each.

For in situ methane decomposition, 100 mg of sample was loaded and heated at 550  C employing a heating rate of 10 K min^−1^ and 100 mL min^−1^ flow of 10% H_2_/He. After 30 min, hydrogen was switched off and 37.5 mL min^−1^ CH_4_ was fed, leading to 30% CH_4_ in He. XRD diffractograms were collected over an angular range from 20° to 55° and a scan rate of 2.3° min^−1^.

H_2_‐TPR studies were performed using the Anton Paar XRK‐900 furnace coupled with a mass spectrometer. 100 mg of the catalysts was heated from room temperature up to 800 °C using a heating rate of 5 °C min^−1^ and 50 mL min^−1^ flow of 4% H_2_ in He.

FTIR spectra have been collected on a Varian Cary 300 UV–vis Spectrophotometer. The spectra were recorded over a wavelength range of 600–4000 cm^−1^ with a data interval of 1 cm^−1^.

Statistical Analysis: Catalytic tests were performed in triplicate, and the mean values along with their standard deviations were reported. Raw TEM images were processed using Gatan Digital Micrograph software. Initial adjustments included the application of automatic brightness and contrast functions to enhance image visibility while preserving structural integrity. No further filtering or modification was applied. Particle size was calculated as the arithmetic mean using the formula: D̄ = (1/N) × Σ Di where D̄ is the average particle diameter, Di is the diameter of the i‐th particle, and N (at least 100) was the total number of particles measured. The standard deviation (SD) was calculated using the formula: SD = sqrt[(1/(N−1)) × Σ (Di − D̄)^2^]. In all cases, statistical analyses were performed using OriginPro software.

### Computational Details—Total Energy Calculations

All total energy (*E_tot_*) calculations were performed within the spin‐polarized density functional theory (DFT) framework using the exchange‐correlation function formulated by Perdew‐Burke‐Ernzerhof (PBE).^[^ ^80^ ^]^ To enhance the description of electron correlation, a Hubbard effective potential U correction of 6.2 eV was applied to the 3d electrons of Ni, following the formalism introduced by Dudarev.^[^ ^81^ ^]^ To maintain the antiferromagnetic ordering of NiO along the (111) direction, a supercell of the conventional cell containing 16 NiO formula units was used. The accuracy of the U parameter was verified by comparing properties like the lattice parameter and band gap calculated by PBE+U against those obtained using the more computationally intensive hybrid HSE06 functional, with data reported in the electronic supporting information.^[^ ^82^ ^]^

To simulate the structure of a 2:1 phyllosilicate with a molecular formula Ni_3_Si_4_O_12_H_2_, a 3‐layer model was employed: two layers of Si_2_O_5_ (one on top and one on the bottom) and one layer of Ni_3_(OH)_2_ in the middle. The Kohn–Sham equations were solved using the projected augmented wave (PAW) method as implemented in the Vienna Ab initio Simulation Package (VASP version 5.4.4).^[^ ^83^ ^]^ A kinetic energy cutoff of 520 eV was used for the plane wave basis set in calculations involving bulks, surfaces, and oxygen/hydroxyl vacancies. For the NiO and Ni_3_Si_4_O_12_H_2_ bulks, 8 × 8 × 8 and 8 × 8 × 6 k‐points grids were used, respectively. Optimized geometries were obtained when the norms of all forces on the atoms were smaller than 0.05 eV Å^−1^ and the total energy convergence criterion was 1.0× 10^−6^ eV.

The NiO(200) and Ni(111) surfaces were modeled using a 2 × 2 slab with four atomic layers, where the two bottom layers were fixed in the bulk geometry, and 16 Å of vacuum was added in the z‐direction between slabs. For the Brillouin zone sampling, 4 × 4 × 1 k‐points were used. This setup also calculates one and two oxygen vacancies on the NiO(200) surface, which presents lower surface energy, Figure S9 (Supporting Information). For Ni_3_Si_4_O_12_H_2_, (001) and (110) facets were explored with a 3‐layer, and the z‐direction vacuum was increased by 16 Å. All atoms were relaxed in the optimization process, and a 7 × 7 × 1 k‐points grid was used. A similar setup was employed for oxygen and hydroxyl vacancies in Ni_3_Si_4_O_12_H_2_(001). All calculations employ dipole correction.^[^ ^84^ ^]^

## Conflict of Interest

The authors declare no conflict of interest.

## Author Contributions

E. G designed the experiments, conducted material synthesis, in situ XRD, H_2_‐TPR, ex situ TEM characterizations, and data analysis, written and revised the first draft, and conceptualized and supervised the project. V. K. O. R. performed the DFT calculations and, together with E. G., the interpretation and revision of the first draft. A. S. B. performed the in situ TEM measurements. V. K. P., supervised by E. G., performed the ex situ XRD measurements and catalytic tests. S. H and I. C. contributed to the discussion and data interpretation. C. D. D supervised the project, acquired funding, and administrated the project. All authors contributed to the discussion and revision of the manuscript.

## Supporting information



Supporting Information

## Data Availability

The data that support the findings of this study are available from the corresponding author upon reasonable request.
