# Supplementary material for: CH4 Decomposition on Nickel Phyllosilicate: Switching from Tip to Base Growth of Carbon Nanotubes
Source: Small. 2025 Jun 4;21(31):2500994. doi: 10.1002/smll.202500994 (PMC12332822; doi:10.1002/smll.202500994)
Supplement: Supplementary file 1 — Supporting Information [file SMLL-21-2500994-s001.pdf]

NANO · MICRO
small

Supporting Information

for *Small*, DOI 10.1002/smll.202500994

CH_4 Decomposition on Nickel Phyllosilicate: Switching from Tip to Base Growth of Carbon Nanotubes

Esteban Gioria, Vivianne K. Ocampo-Restrepo, Anton Simon Bjørnlund, Verdande Kim Pedersen, Stig Helveg, Ib Chorkendorff and Christian Danvad Damsgaard**

Supporting Information: In-situ Investigations of CH_4 Decomposition on Nickel Phyllosilicate: Switching from Tip to Base Growth of Carbon Nanotubes

Esteban Gioria[1*], Vivianne K. Ocampo-Restrepo[1], Anton Simon Bjørnlund[2], Verdande Kim Pedersen[1], Stig Helveg[1], Ib Chorkendorff[1], Christian D. Damsgaard[1,2,3*]

[1]DTU Physics, Technical University of Denmark, Kongens Lyngby, DK-2800, State, Denmark.
[2]Center for Visualizing Catalytic Processes, VISION, Technical University of Denmark, Kongens Lyngby, DK-2800, Denmark.
[3]DTU Nanolab, Technical University of Denmark, Kongens Lyngby, DK-2800, Denmark.

*Corresponding author(s). E-mail(s): egagi@dtu.dk; cdda@dtu.dk;

List of Figures

S1 **Scanning electron microscopy studies of calcined Ni/SiO_2 and Ni-PS: a) secondary electrons; b)backscattered electrons. Scale bar 500 nm.** . . . 3
S2 **a) Relative ratio of reduced Ni to total Ni and b) Crystallite size versus temperature during the in-situ XRD reduction studies.** . . . 4
S3 **Diameter distribution of carbon nanotubes formed at 550 °C.** 5
S4 **TEM of Ni-PS-Red after methane decomposition at 550 °C.** . . 6
S5 **Total energies of antiferromagnetic (AF) non-order NiO as a function of lattice constant.** . . . 7
S6 **AF ordering along (111) direction in NiO bulk. The green and blue planes represent Ni atoms' up and down spin alignments, respectively. Gray and red colors represent Ni and O atoms, respectively.** . . . 8
S7 **Structure of $Ni_3Si_4O_{12}H_2$ composed of two layers of Si_2O_5 (one on top and one on the bottom) and one layer of $Ni_3(OH)_2$ in the middle.** . . . 9
S8 **Relative energies of $Ni_3Si_4O_{12}H_2$ as a function of total magnetic moment: The lowest energy is achieved at a total magnetic moment of 6, which aligns with the magnetic nature of this material [1].** . . . 10
S9 **Optimized surfaces for NiO(200), left, and NiO(111), right side, along with their surface energies.** . . . 12
S10 **Optimized surfaces for $Ni_3Si_4O_{12}H_2$(001) left, and $Ni_3Si_4O_{12}H_2$(110), right side, along with their surface energies.** . . 13

Contents

1 Additional experimental information: 3

2 Additional computational details: 7
2.1 Benchmarking antiferromagnetic (AF) ordering in NiO bulk: 8
2.2 Nickel Phyllosilicate 2:1 ($Ni_3Si_4O_{12}H_2$) . . . 9

3 DFT study about Ni−O bond reducibility of 2:1 Ni-phyllosilicate ($Ni_3Si_4O_{12}H_2$) vs. NiO 11
3.1 Surfaces from NiO . . . 12
3.2 Surfaces from Ni phyllosilicate 2:1 ($Ni_3Si_4O_12H_2$): . . . 13

4 Thermodynamic analysis of oxygen vacancies formation 14

S1 Additional experimental information:

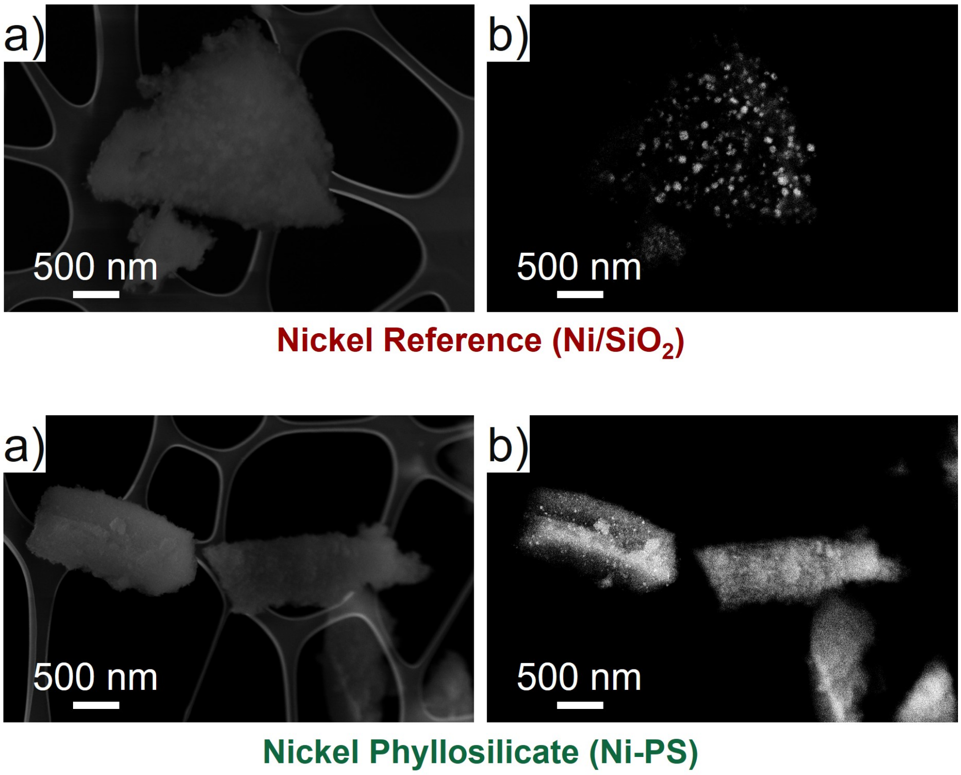

Fig. S1: Scanning electron microscopy studies of calcined Ni/SiO_2 and Ni-PS: a) secondary electrons; b)backscattered electrons. Scale bar 500 nm.

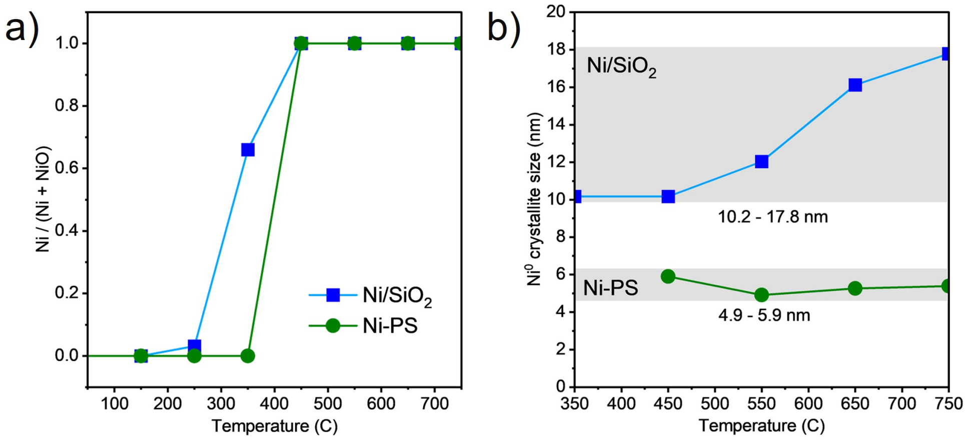

Fig. S2: **a) Relative ratio of reduced Ni to total Ni and b) Crystallite size versus temperature during the in-situ XRD reduction studies.**

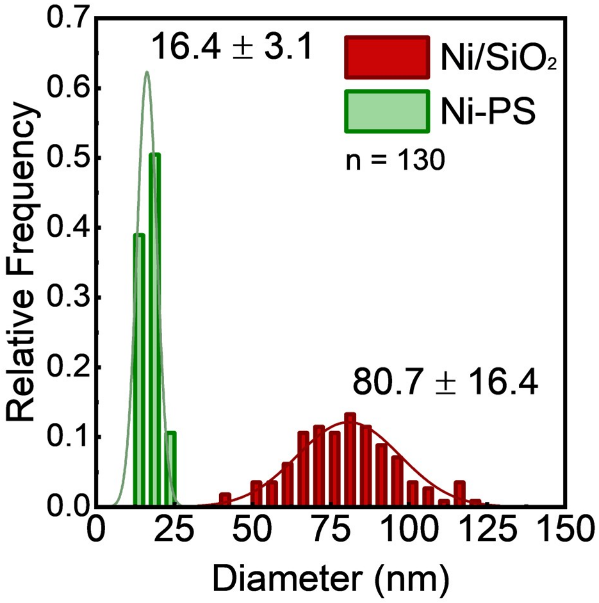

Fig. S3: **Diameter distribution of carbon nanotubes formed at 550 °C.**

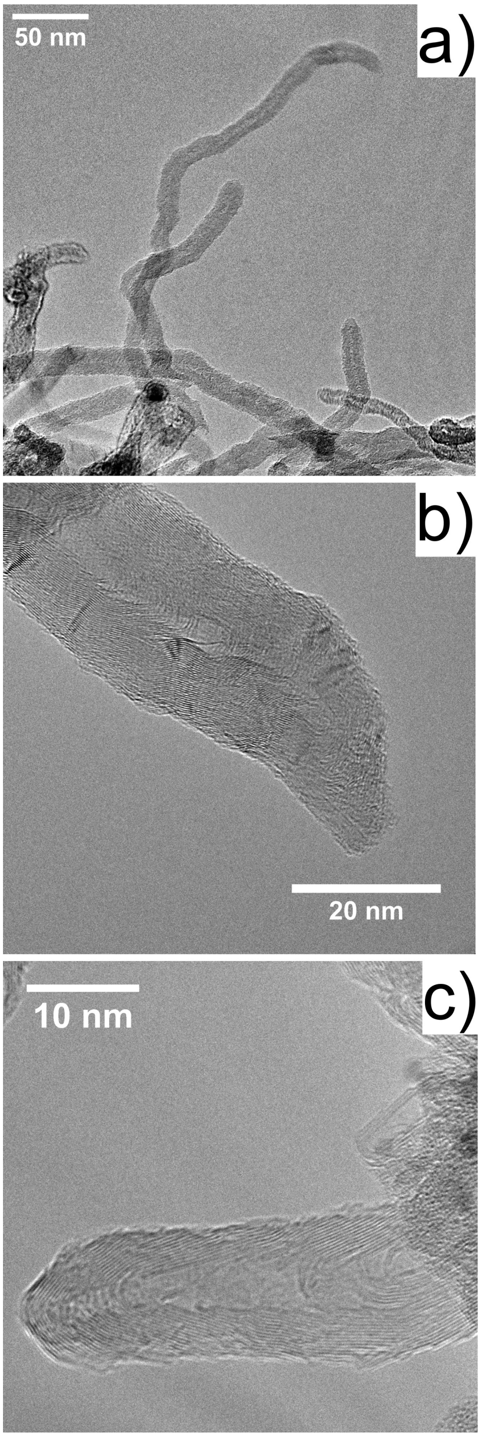

Fig. S4: TEM of Ni-PS-Red after methane decomposition at $550\,^{\circ}\mathrm{C}$**.**

S2 Additional computational details:

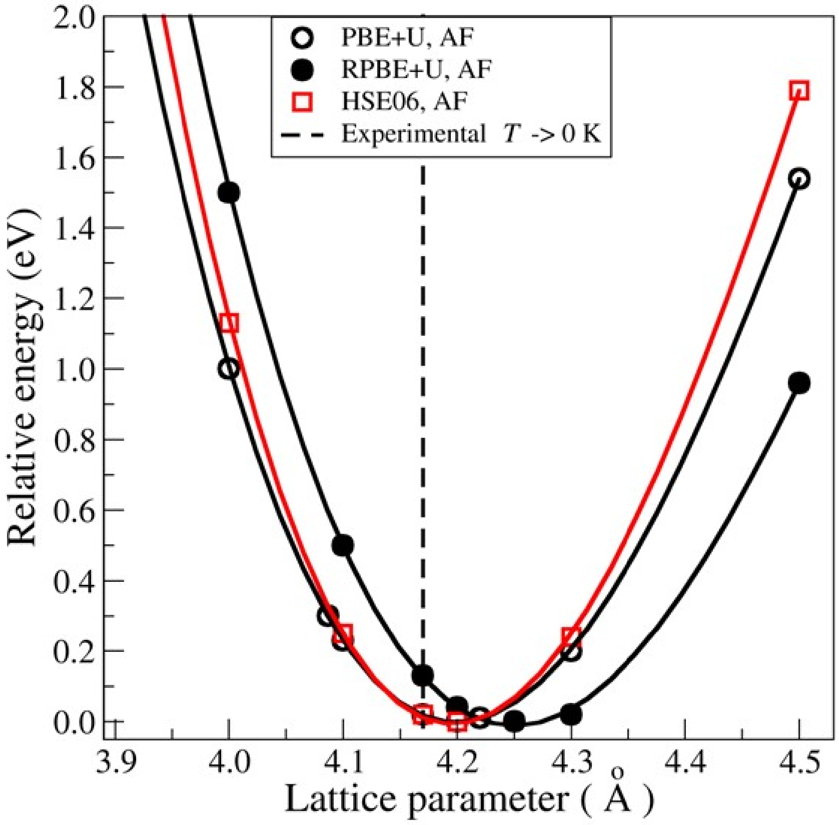

Fig. S5: **Total energies of antiferromagnetic (AF) non-order NiO as a function of lattice constant.**

All energies are plotted relative to the energy at the equilibrium lattice parameter. The vertical dashed line indicates the experimental equilibrium lattice constant, 4.17 Å [2]. We use a conventional cell with four NiO formula units and $6 \times 6 \times 6$ k-points grid for PBE+U and RPBE+U functionals, while the HSE06 calculations use $4 \times 4 \times 4$ k-points grid.

S2.1 Benchmarking antiferromagnetic (AF) ordering in NiO bulk:

Table S1: **Comparison of antiferromagnetic (AF) NiO properties: theoretical results obtained using PBE+U (with U =** $6.2\,\text{eV}$**) and the HSE**06 **functionals are compared with previously reported experimental data [3][4][5][6]. To guarantee the AF order along (111) direction, we employ a supercell** $(2\times2\times1)$ **with** 16 **NiO formula units. A k-points grid of** $4\times4\times8$ **and** $2\times2\times4$ **are used for PBE+U and HSE**06**, respectively.**

**Functional**	**AF Ordering**	**Relative Energy** (eV)	**Magnetic Moment of Ni ion** (μ_B)	**Band Gap** (eV)	**Lattice Parameter** (Å)
PBE + U	Non-order	0.40	1.80	2.74	4.20
	111	0.00	1.75	3.27	4.19
HSE06	Non-order	0.60	1.72	3.22	4.19
	111	0.00	1.69	4.03	4.18
Experimental	111		1.81 - 2.20	3.40 - 4.30	4.17

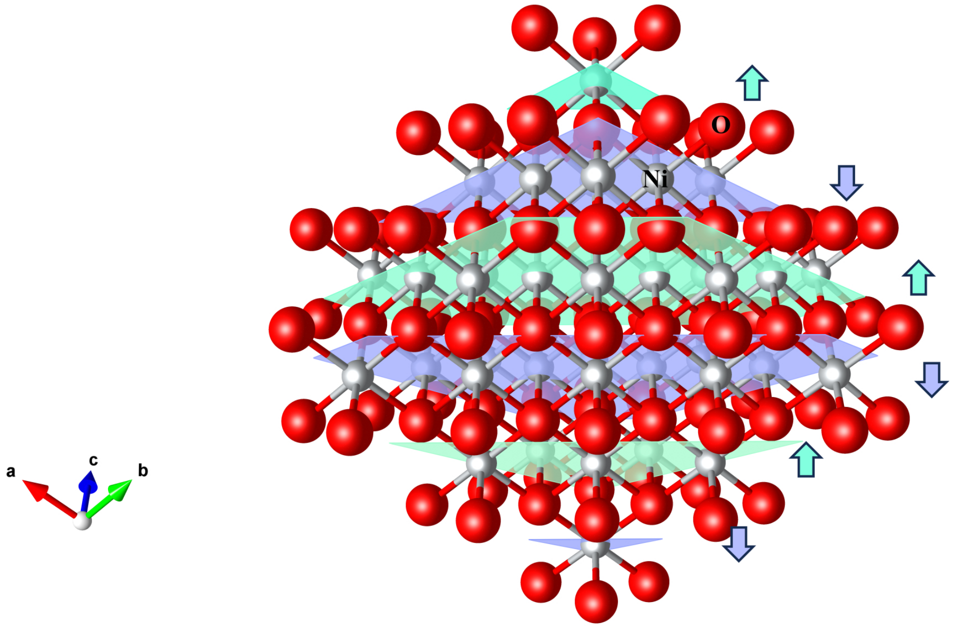

Fig. S6: **AF ordering along (111) direction in NiO bulk. The green and blue planes represent Ni atoms' up and down spin alignments, respectively. Gray and red colors represent Ni and O atoms, respectively.**

S2.2 Nickel Phyllosilicate 2:1 ($Ni_3Si_4O_{12}H_2$)

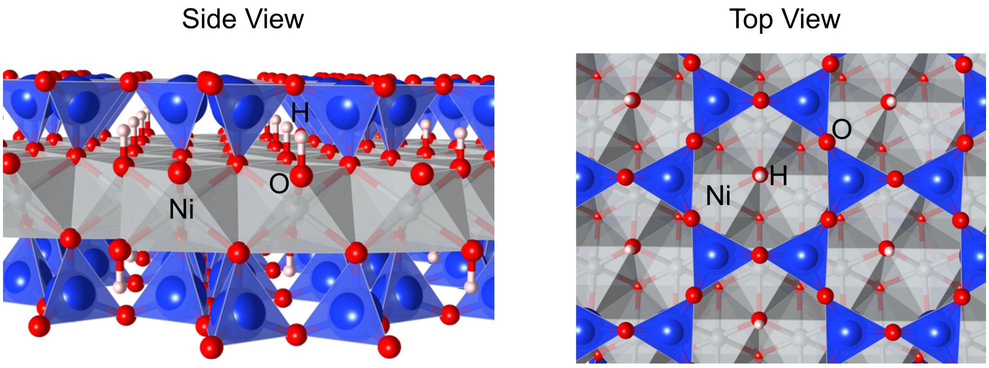

Fig. S7: **Structure of $Ni_3Si_4O_{12}H_2$ composed of two layers of Si_2O_5 (one on top and one on the bottom) and one layer of $Ni_3(OH)_2$ in the middle.**

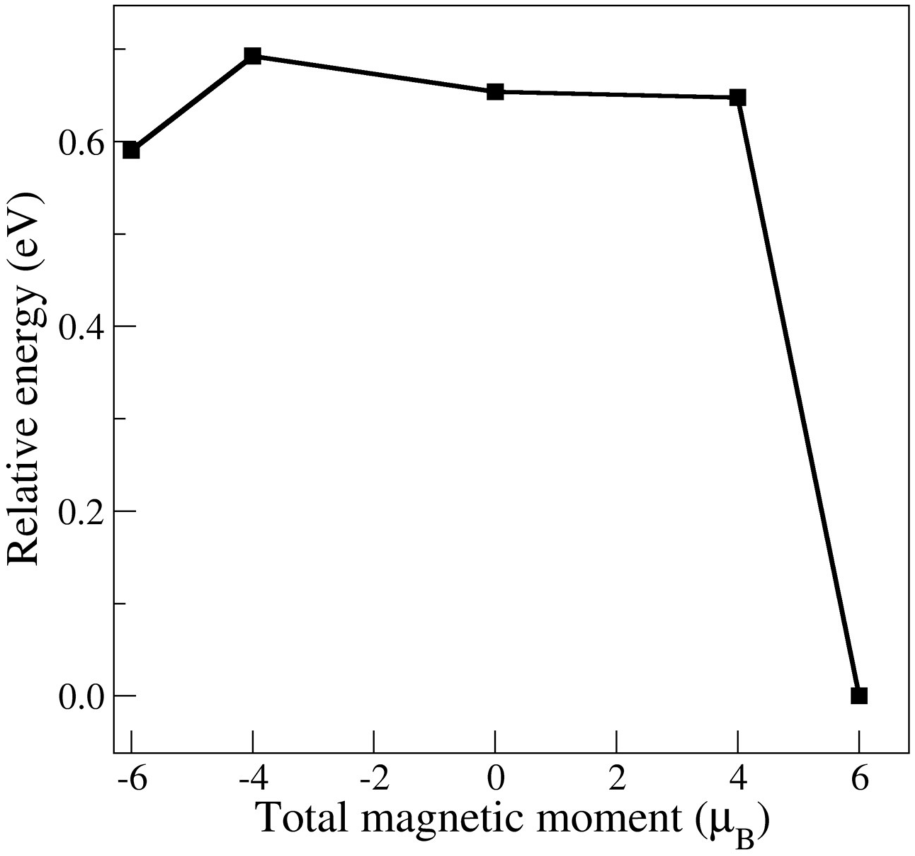

Fig. S8: **Relative energies of $Ni_3Si_4O_{12}H_2$ as a function of total magnetic moment: The lowest energy is achieved at a total magnetic moment of 6, which aligns with the magnetic nature of this material [1].**

S3 DFT study about Ni−O bond reducibility of 2:1 Ni-phyllosilicate ($Ni_3Si_4O_{12}H_2$) vs. NiO

We want to understand the differences in the Ni−O bond between the precursors used to synthesize Ni/SiO_2-Red and Ni-PS-Red. For Ni/SiO_2, the reduction peak related to the formation of Ni nanoparticles appears at 350 °C in the In-Situ XRD under hydrogen. While this peak is observed at higher temperatures, at 450 °C, for Ni-PS. Based on the initial spectra at room temperature (RT) before heating, the precursor for Ni/SiO_2-Red appears to be a NiO-based material. We use NiO as a model to study the reducibility of the precursor for the synthesis of Ni/SiO_2-Red by DFT. For the Ni-PS-Red, we consider a 2:1 Ni-phyllosilicate ($Ni_3Si_4O_{12}H_2$) as a model for the precursor. We use the bulk structures of NiO and $Ni_3Si_4O_{12}H_2$(Figures S9 and S10), to explore the most stable surfaces for both cases by calculating their surface energies (E_{surf}) as follows:

$$E_{surf} = \frac{1}{2}A(E_{tot}^{surf} - N * E_{tot}^{bulk}), \tag{1}$$

where,

- A: is the area obtained from the x and y-axis measurements in the optimized structure used for the surface calculation (Å^2).
- E_{tot}^{surf}: is the total energy for the surface (eV).
- N: represents the number of formula units contained in the structure of the surface calculation.
- E_{tot}^{bulk}: is the total energy of the bulk with one formula unit (eV).

The surfaces with the smaller surface energies are the most favored. We then use the most favored surfaces to study oxygen vacancies, which provide insights into how thermodynamically favored the reduction is.

S3.1 Surfaces from NiO

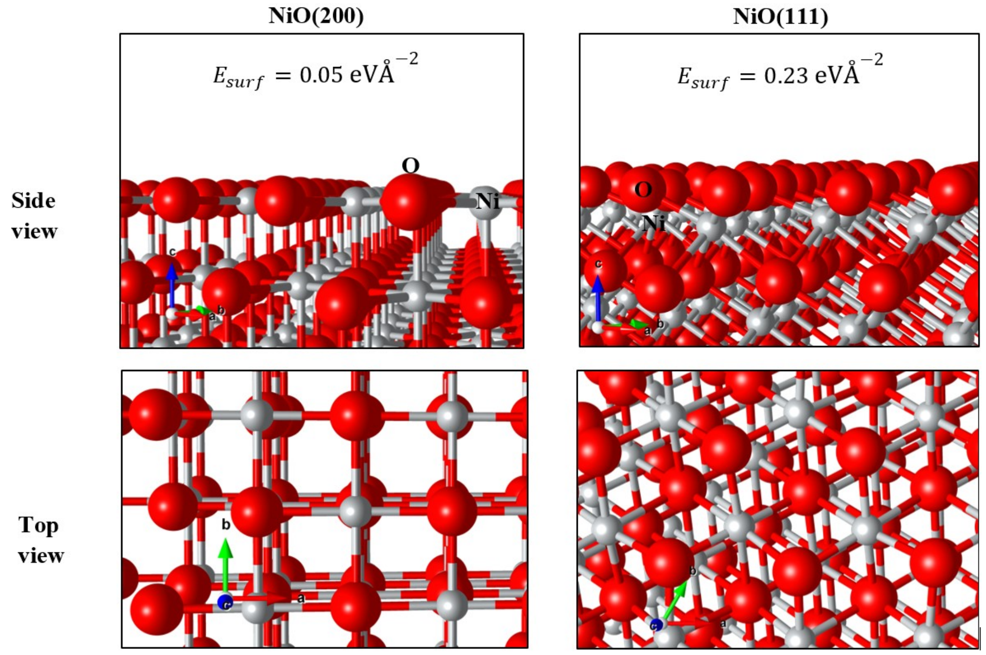

Fig. S9: Optimized surfaces for NiO(200), left, and NiO(111), right side, along with their surface energies.

S3.2 Surfaces from Ni phyllosilicate 2:1 ($Ni_3Si_4O_12H_2$):

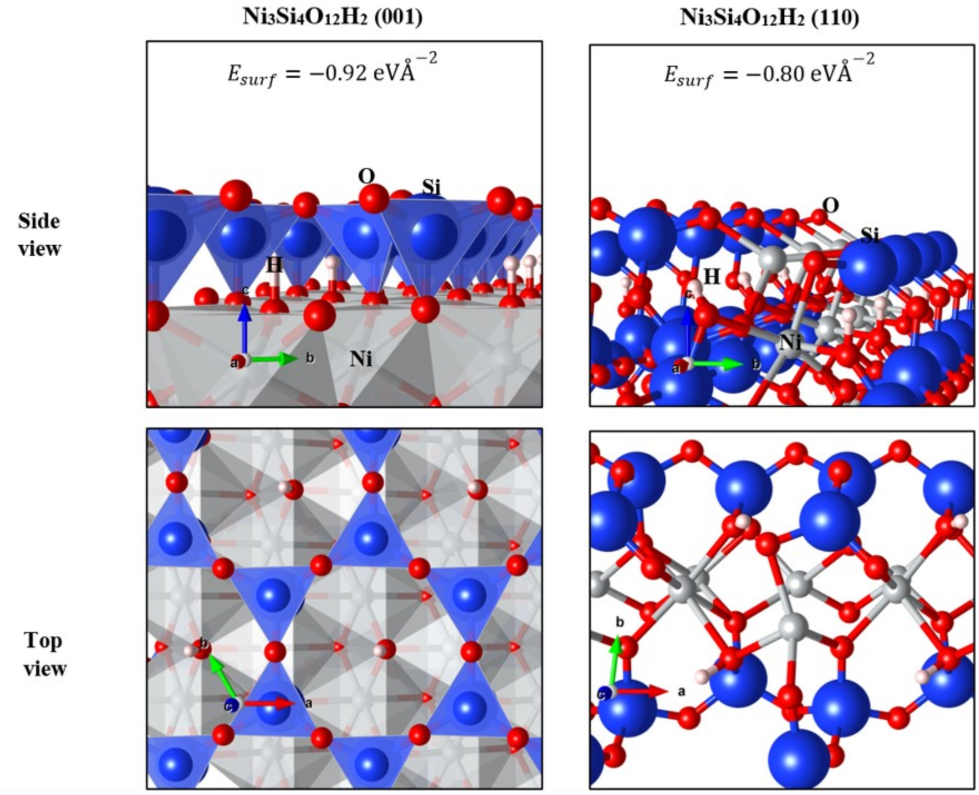

Fig. S10: **Optimized surfaces for $Ni_3Si_4O_{12}H_2$(001) left, and $Ni_3Si_4O_{12}H_2$(110), right side, along with their surface energies. .**

S4 Thermodynamic analysis of oxygen vacancies formation

Table S2: Thermodynamic properties calculated at different temperatures and 0.1 bar for H_2(g) and H_2O (g) within the ideal gas limit as implemented in the thermochemistry module available in the atomistic simulation environment package. E^{ZPE} denotes the zero-point energy, C_pdT indicates the heat capacities, S represents the entropy, and T is the temperature.

Temperature (°C)	Properties (eV)	H_2(g)	H_2O(g)
	E^{ZPE}	0.27	0.56
25	C_pdT	0.03	0.03
	TS	0.46	0.64
100	C_pdT	0.03	0.03
	TS	0.60	0.83
150	C_pdT	0.04	0.04
	TS	0.70	0.97
250	C_pdT	0.05	0.05
	TS	0.90	1.23
350	C_pdT	0.05	0.05
	TS	1.10	1.51
450	C_pdT	0.06	0.06
	TS	1.31	1.79
550	C_pdT	0.07	0.07
	TS	1.53	2.08
650	C_pdT	0.08	0.08
	TS	1.75	2.38
750	C_pdT	0.09	0.09
	TS	1.97	2.68

Table S3: Change in energy for the formation of the oxygen vacancies per H_2O(g) produced,ΔE_f.

	NiO(200)	
	$n = 1$	$n = 2$
ΔE_f per H_2O (eV)	2.10	1.87
	nO-$Ni_3Si_4O_{10}(OH)_2$(001)	
	$n = 1$	$n = 2$
ΔE_f per H_2O (eV)	2.90	2.80
	nOH-$Ni_3Si_4O_{10}(OH)_2$(001)	
	$n = 1$	$n = 2$
ΔE_f per H_2O (eV)	1.78	1.64

References

[1] Richard-plouet, M., Vilminot, S.: Magnetic properties of two-dimensional triangular arrays of Ni ions in nickel phyllosilicates. Journal of Materials Chemistry **8**(1), 131–137 (1998) https://doi.org/10.1039/a702985c

[2] Bartel, L.C., Morosin, B.: Exchange Striction in NiO. Physical Review B **3**(3), 1039–1043 (1971) https://doi.org/10.1103/PhysRevB.3.1039

[3] Tran, F., Blaha, P., Schwarz, K., Novák, P.: Hybrid exchange-correlation energy functionals for strongly correlated electrons: Applications to transition-metal monoxides. Physical Review B **74**(15), 155108 (2006) https://doi.org/10.1103/PhysRevB.74.155108

[4] Sawatzky ', G.A., Allen, J.W.: PHYSICAL REVIEW LETTERS Magnitude and Origin of the Band Gap in Nio. Technical report

[5] Irwin, M.D., Buchholz, D.B., Hains, A.W., Chang, R.P.H., Marks, T.J.: p-Type semiconducting nickel oxide as an efficiency-enhancing anode interfacial layer in polymer bulk-heterojunction solar cells. Technical report (2008). https://doi.org/10.1073Θpnas.0711990105 . www.pnas.org/cgi/content/full/

[6] Patil, P.S., Kadam, L.D.: Preparation and characterization of spray pyrolyzed nickel oxide (NiO) thin films. Applied Surface Science **199**(1-4), 211–221 (2002) https://doi.org/10.1016/S0169-4332(02)00839-5
